# A novel dilated contextual attention module for breast cancer mitosis cell detection

**DOI:** 10.3389/fphys.2024.1337554

**Published:** 2024-01-25

**Authors:** Zhiqiang Li, Xiangkui Li, Weixuan Wu, He Lyu, Xuezhi Tang, Chenchen Zhou, Fanxin Xu, Bin Luo, Yulian Jiang, Xingwen Liu, Wei Xiang

**Affiliations:** ^1^ Key Laboratory of Electronic and Information Engineering, State Ethnic Affairs Commission, Southwest Minzu University, Chengdu, Sichuan, China; ^2^ School of Computer Science and Technology, Harbin University of Science and Technology, Harbin, China; ^3^ Chongqing Key Laboratory of Computational Intelligence, Chongqing University of Posts and Telecommunications, Chongqing, China; ^4^ Sichuan Huhui Software Co., LTD., Mianyang, Sichuan, China

**Keywords:** mitosis detection, mitotic count, dilated attention, whole slide image, multi-stage deep learning

## Abstract

**Background and object:** Mitotic count (MC) is a critical histological parameter for accurately assessing the degree of invasiveness in breast cancer, holding significant clinical value for cancer treatment and prognosis. However, accurately identifying mitotic cells poses a challenge due to their morphological and size diversity.

**Objective:** We propose a novel end-to-end deep-learning method for identifying mitotic cells in breast cancer pathological images, with the aim of enhancing the performance of recognizing mitotic cells.

**Methods:** We introduced the Dilated Cascading Network (DilCasNet) composed of detection and classification stages. To enhance the model’s ability to capture distant feature dependencies in mitotic cells, we devised a novel Dilated Contextual Attention Module (DiCoA) that utilizes sparse global attention during the detection. For reclassifying mitotic cell areas localized in the detection stage, we integrate the EfficientNet-B7 and VGG16 pre-trained models (InPreMo) in the classification step.

**Results:** Based on the canine mammary carcinoma (CMC) mitosis dataset, DilCasNet demonstrates superior overall performance compared to the benchmark model. The specific metrics of the model’s performance are as follows: F1 score of 82.9%, Precision of 82.6%, and Recall of 83.2%. With the incorporation of the DiCoA attention module, the model exhibited an improvement of over 3.5% in the F1 during the detection stage.

**Conclusion:** The DilCasNet achieved a favorable detection performance of mitotic cells in breast cancer and provides a solution for detecting mitotic cells in pathological images of other cancers.

## 1 Introduction

Breast cancer is among the most common malignancies, with a high incidence and mortality rate among women worldwide (Xu et al., 2023). Histopathological image analysis has long been regarded as the “gold standard” in cancer diagnosis and prognosis evaluation (Gurcan et al., 2009). The identification of molecular quantities and features within patients’ tumors is crucial for the clinical treatment and prognosis assessment of cancer patients (Dai et al., 2023). Within histopathological image analysis, the mitotic count is recognized as a critical histological parameter for diagnosing and grading cancer (Cree et al., 2021). However, the current MC still relies on manual counting of mitotic figures through an optical microscope (Bertram et al., 2020), and even pathologists only maintain moderate consistency in identifying mitotic cells (Ibrahim et al., 2021).

To achieve automated mitotic detection to assist pathologists in diagnosis, traditional machine learning approaches depend on prior knowledge, employing carefully designed handcraft feature extractors to process features and integrate various machine learning classifiers for mitotic cell identification (Lu and Mandal, 2014; Paul and Mukherjee, 2015; Mathew et al., 2021). Although manual feature extraction contributes to the comprehension of mitotic cell characteristics, their generalization performance across large-scale datasets is constrained.

With the continuous advancement of deep learning, convolutional neural networks (CNNs) have provided new solutions for mitotic cell detection (Lecun et al., 2015; Lin et al., 2016; Huang et al., 2017). Concurrently, the availability of publicly accessible datasets featuring expert-annotated images of mitotic cells, such as ICPR MITOS-2012 (Ludovic et al., 2013), AMIDA 2013 (Veta et al., 2015), ICPR MITOS-ATYPIA-2014 (MITOS-ATYPIA-14., 2014), and TUPAC 2016 (Veta et al., 2019), has facilitated the application of deep learning methods in mitotic cell detection. However, these datasets only contain annotated mitotic images corresponding to High Power Fields (HPF) (Bertram et al., 2020) in hotspots and lack annotations for most areas of whole slide images (WSI). Recently, two extensive WSI datasets with annotated mitotic cells have been introduced: the canine cutaneous mast cell tumor (CCMCT) dataset (Bertram et al., 2019) and the canine mammary carcinoma (CMC) dataset (Aubreville et al., 2020). These datasets enable automatic mitotic detection models to learn from a more extensive collection of mitotic images and their contextual information (Bertram et al., 2019).

Previous studies have directly applied deep learning models for the recognition of mitotic cells (Cireşan et al., 2013; Zerhouni et al., 2017), but these existing methods lack adequate domain adaptability. Currently, mitotic recognition methods typically utilize multi-stage models that integrate various tasks including detection, segmentation, and classification (Li et al., 2018; Alom et al., 2020), which perform better than single models. The diverse and intricate morphological features of mitotic cells across different cell cycle phases result in significant heterogeneity. Moreover, mitotic cells are often sparsely distributed and can be easily mistaken for other cell types, such as apoptotic cells and densely packed nuclear cells, when compared to normal cells (Ibrahim et al., 2022). Currently, multi-stage mitotic detection and classification models have not specifically focused on the impact of feature extraction and application on model performance.

We propose a two-stage Dilated Cascading Network (DilCasNet) to improve the performance of mitosis detection. In the mitosis cell detection stage, inspired by the Extended Contextual Attention (DiNA) (Hassani and Shi, 2022) and Polar Attention Network (PolarNet) (Wei et al., 2022), we propose a novel attention module, namely, Dilated Contextual Attention (DiCoA), and combine it with the Feature Pyramid Network (FPN) (Lin et al., 2016) of the Cascade RCNN (Cai and Vasconcelos, 2017) detection network to enhance the detection performance of mitosis cells. In the classification stage, we integrate the EfficientNet-B7 (Tan and Le, 2019) and VGG16 (Simonyan and Zisserman, 2015) pre-trained models to enhance the model’s classification performance. The main contributions of this study are as follows:

(1) Introducing DiCoA, a sparse global attention module based on the self-attention mechanism, which achieves a larger receptive field by sparsifying keys and values, enabling the model to benefit in the challenging task of mitotic cell detection with complex morphologies. Experimental evidence demonstrates that incorporating DiCoA into the FPN of the Cascade R-CNN detection network reduces false positive predictions and enhances the model’s performance in recognizing mitotic cells.
(2) To enhance the feature extraction of mitotic cells by the classification model, we integrate the EfficientNet-B7 and VGG16 pre-trained models (InPreMo), further improving the performance of the mitotic cell detection model by combining various CNN pre-trained models.

## 2 Related work

Many automated algorithms for mitosis cell detection have been proposed to assist pathologists in diagnosis. In the early stages, manual design and feature selection methods were typically employed to achieve automated detection (Mathew et al., 2021). The entire process is generally divided into two steps: First, restrict the detection scope to specific candidate regions selected for segmentation. Subsequently, directly extract features from the image, including texture, statistical, and morphological features (Irshad et al., 2013; Paul and Mukherjee, 2015; Nateghi et al., 2017), or extract features from different color spaces (Irshad et al., 2014b; 2014a; Lu and Mandal, 2014). The extracted features are then used to develop decision trees, random forests (RF), support vector machines (SVM) (Udousoro, 2020), and other classifiers to distinguish non-mitotic cells from mitotic cells in pathological slides. These methods have demonstrated competitive performance on datasets such as ICPR MITOS-2012, AMIDA 2013, and ICPR MITOS-ATYPIA-2014. However, manual feature extraction primarily relies on handcrafted feature extractors, making the process labor-intensive and challenging to extract deep abstract features.

With the development of deep learning, CNNs have demonstrated excellent capabilities in automatic feature extraction and learning and have achieved significant performance in tasks such as image classification, object detection, and semantic segmentation (Lecun et al., 2015; Ren et al., 2015; Weng and Zhu, 2015). Consequently, CNNs have found widespread applications in medical image processing (Tran et al., 2021). In the mitosis detection research, some experts and scholars choose the recently popular deep convolutional neural networks for automatic mitosis detection. Employing deep learning algorithms, pixel-wise classifiers have been developed to compute the probability of each pixel being associated with a mitotic event (Cireşan et al., 2013; Zerhouni et al., 2017). These approaches demonstrate a high level of accuracy. To further augment the model’s capacity for extracting mitotic cell features, multi-stage deep learning approaches (Li et al., 2018; Alom et al., 2020) were adopted, combining detection, segmentation, and classification tasks to develop two-stage and three-stage models. Similar to existing studies, our approach also adopts a two-stage method for mitotic classification detection. The utilization of multiple classifiers (Wang et al., 2014; Beevi et al., 2017; Mahmood et al., 2020), combined with handcrafted features, segmentation, or detection methods, achieved mitosis detection in a cascaded manner, further strengthening the model’s capability in feature extraction and mitotic cell recognition. These methods have all demonstrated various degrees of performance improvement on the ICPR MITOS-2012 and MITOS-ATYPIA-2014 datasets. However, these two datasets have limited images and samples, and most of the WSI regions lack images and annotations, which poses challenges for model training. In accordance with recommendations from existing studies (Aubreville et al., 2020), we utilized a larger-scale CMC dataset for model training and evaluation.

Due to the diverse shapes of mitotic cells, attention modules are widely considered effective for better feature extraction from data (Brauwers and Frasincar, 2022). Hu et al. (2020) introduced Squeeze-and-Excitation Networks (SENet), which construct interdependencies among feature channels through weighted operations to enhance model expressiveness. Regarding spatial information processing, Huang et al. (2018) proposed the Criss-Cross Network (CCNet) to help the network obtain contextual information from the image, allowing each pixel to perceive its relevance to the entire image. To simultaneously focus on channel and spatial information, Woo et al. (2018) introduced the Convolutional Block Attention Module (CBAM), which combines channel and spatial attention, maintaining a small overhead while improving the model’s focus on spatial and channel features. Multiple studies have demonstrated that introducing attention modules effectively enhances the model’s feature extraction capability. However, these classical attention mechanisms are not specifically designed for mitotic detection and cannot fully leverage the potential of attention mechanisms to enhance model performance in mitotic classification detection. Therefore, we have devised a novel attention mechanism to address this purpose. Simultaneously, transfer learning methods (Pan and Yang, 2010) have been widely applied in various tasks to alleviate the issues of training network models requiring time and limited training data, which are of great significance for automated mitotic cell detection research. These methods have positive implications for enhancing the performance of automated detection of mitotic cells.

## 3 Materials and methods

### 3.1 Materials

#### 3.1.1 CMC dataset

This study utilized a dataset of 21 WSIs for CMC (Aubreville et al., 2020), which encompassed three different modes of annotations: Manually Expert Labeled (MEL), Object-Detection Augmented and Expert Labeled (ODAEL), and Clustering and Object-Detection Augmented and Expert Labeled (CODAEL). To facilitate comparison with prior research (Piansaddhayanaon et al., 2023), we followed the methodology presented in the previous study, using the CODAEL annotations for training and testing, with 14 of the WSIs in the training set and the remaining 7 in the test set. Detailed information on this dataset is provided in Supplementary Data SA.1.1.

#### 3.1.2 CCMCT dataset

This study conducts generalization validation using the CCMCT dataset, which comprises 32 WSIs. The dataset includes three different annotation methods for various categories: Manually Expert Labeled (MEL), Hard-Example Augmented Expert Labelled (HEAEL), and Object-Detection Augmented Expert Labelled (ODAEL). To facilitate comparison with prior research (Bertram et al., 2019), we performed testing on a test set containing 11 WSIs. For detailed information on this dataset, please refer to Supplementary Data SA.1.2.

### 3.2 Methods

**FIGURE 1:**
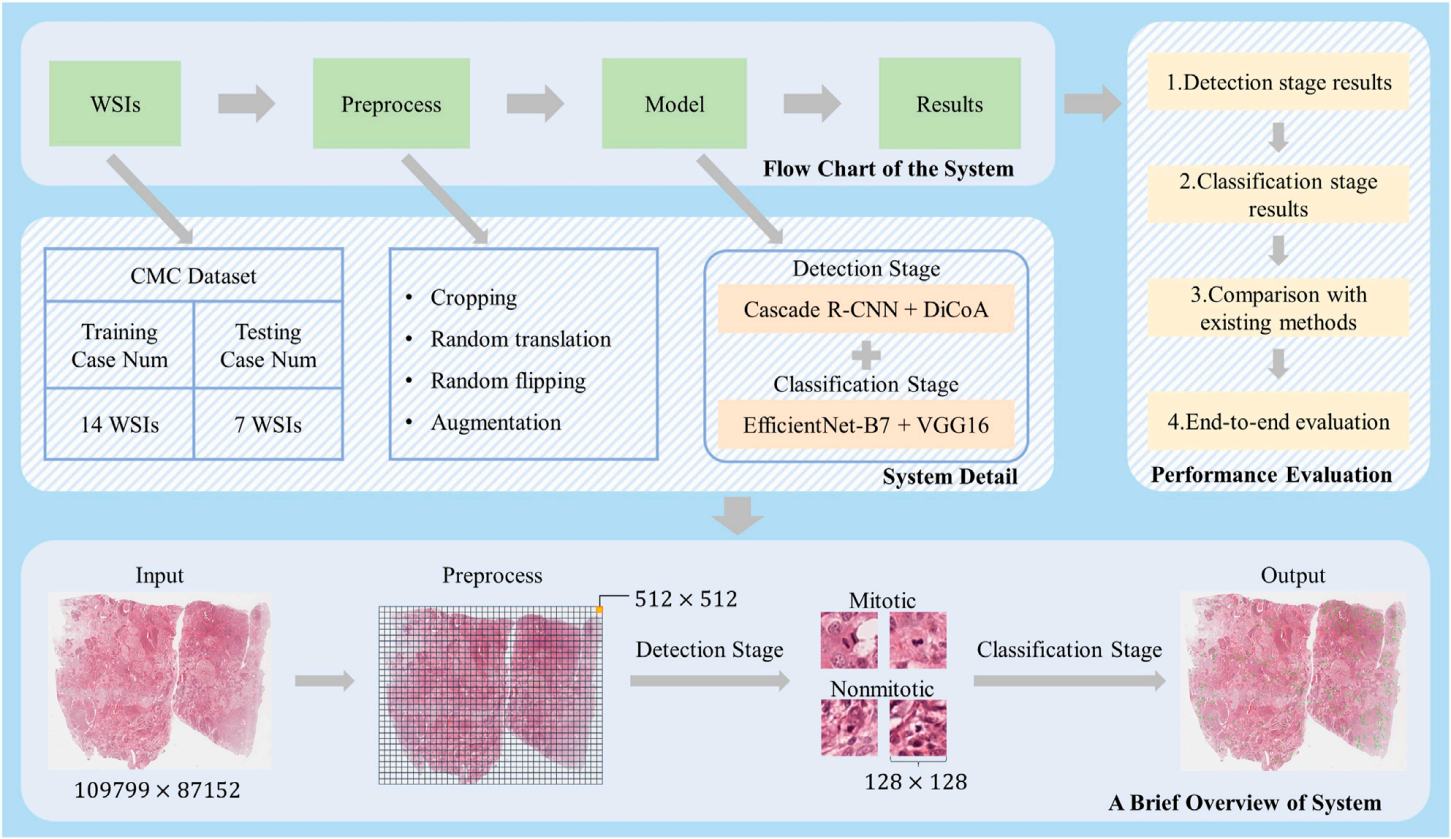
Overall Study Design. At the top, the flowchart of our method is presented; the middle section provides detailed implementation steps; after preprocessing of the massive WSIs, different-sized images for the detection and classification stages of model training are generated; on the right, our method’s performance is demonstrated in various aspects; at the bottom, a brief overview of how our method processes WSI is outlined.

Figure 1 illustrates the overall workflow of the mitosis detection model DilCasNet. Large WSIs, after undergoing preprocessing steps such as cropping, are input into the model to detect mitotic cells. The model is primarily divided into two stages: the mitotic detection stage utilizing Cascade R-CNN with DiCoA attention and the mitotic cell classification stage incorporating pre-trained models, EfficientNet-B7 and VGG16.

#### 3.2.1 DiCoA module

**FIGURE 2:**
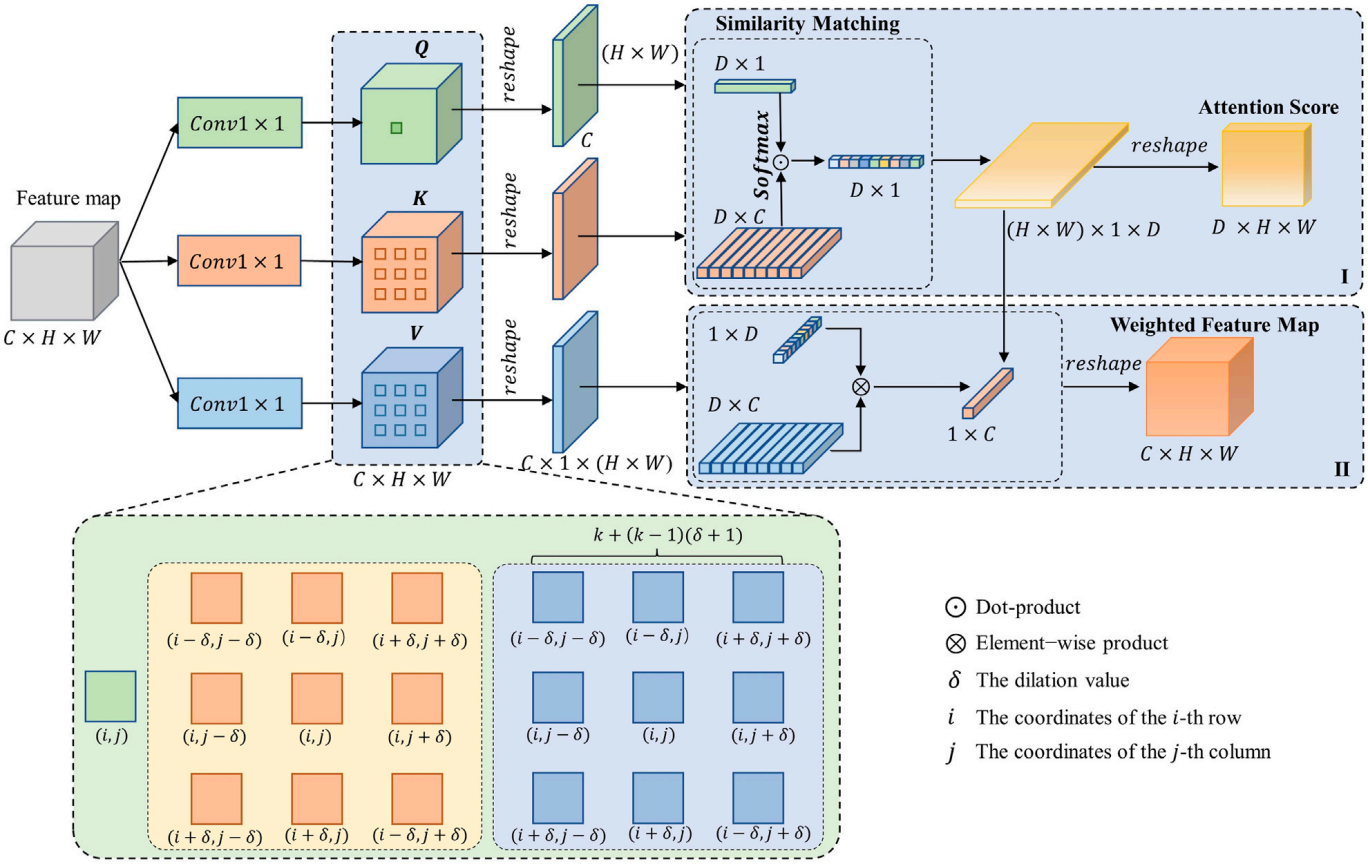
The structural diagram of DiCoA, where C , H , and W represent the number of channels, height, and width of the input feature map, respectively.

The design of DiCoA is illustrated in Figure 2. In the first step, DiCoA obtains the attention score matrix of the dilation contextual by calculating the self-attention within the neighborhood of the feature expansion interval, as shown in Figure 2 (I). Subsequently, DiCoA further generates new feature maps by weighting the attention scores in different directions within the interval neighborhood region, as depicted in Figure 2 (II).

Calculating the attention scores for the dilated contextual of feature maps: Given the input feature map $x\in R^{C\times H\times W}$ , projections of the feature map queries ( $Q\in R^{C\times H\times W}$ ), keys ( $K\in R^{C\times H\times W}$ ), and values ( $V\in R^{C\times H\times W}$ ) are obtained through 2D convolution. The formula for the matrix of attention scores for the dilated contextual, $DPS\in R^{C\times H\times W}$ , as shown in Formula 1.

$${DPS}_{D,i,j}^{\delta }=softmax\left(Q_{c,i,j}⊙K_{c,\rho_{D}^{\delta }\left(i,j\right)}^{T}\right)$$ (1)

Where i and j denote the coordinates of the i -th row and j -th column in the feature map. $Q_{c,i,j}$ represents the nearest neighbor query for the pixel at coordinates $\left(i,j\right)$ . ⊙ denotes the dot product operation. D represents the total number of pixels in a neighborhood of size k ; we set the k to 3 and the D to 9. $\delta \in \left[1,k\right]$ represents the dilation value; we set the δ to 1. $\rho_{D}^{\delta }\left(i,j\right)\in N^{D\times 2}$ denotes the positions of neighbors in the δ -dilated neighborhood of the $\left(i,j\right)$ -th pixel (see Supplementary Data SA.2.1). $K_{c,\rho_{D}^{\delta }\left(i,j\right)}$ represents the key for the $\left(i,j\right)$ -th pixel in the δ -dilated neighborhood of size k . In addition, Supplementary Data SA.2.2 provides the update method for attention scores in the network.

Updating the feature maps of the network: The dot product operation between Q and K results an dilated contextual attention score matrix, DPS , of size $\left(H\times W\right)\times 1\times D$ . After resizing, it becomes a D×H×W matrix. Finally, matrix operation between DPS and V results in the weighted feature map $y\in R^{C\times H\times W}$ with dimensions C×H×W , as depicted in Formula 2.

$$y_{c,i,j}=norm\left(\sum_{n=1}^{D}{DPS}_{n,i,j}^{\delta }\times V_{c,i,j}^{\left(D,\delta \right)}+x\right)$$ (2)

Where c represents the channel size of the feature map. norm denotes the data normalization method. $V_{c,i,j}^{\left(D,\delta \right)}$ represents the value projection of the δ -dilated neighborhood of size k for the $\left(i,j\right)$ -th pixel, which can be expressed by Formula 3.

$$V_{c,i,j}^{\left(D,\delta \right)}={\left[\begin{array}{cc} V_{c,\rho_{1}^{\delta }\left(i,j\right)}^{T} & \begin{array}{cc} V_{c,\rho_{2}^{\delta }\left(i,j\right)}^{T} & \begin{array}{cc} \cdots & V_{c,\rho_{D}^{\delta }\left(i,j\right)}^{T} \end{array} \end{array} \end{array}\right]}^{T}$$ (3)

#### 3.2.2 Network architecture

**FIGURE 3:**
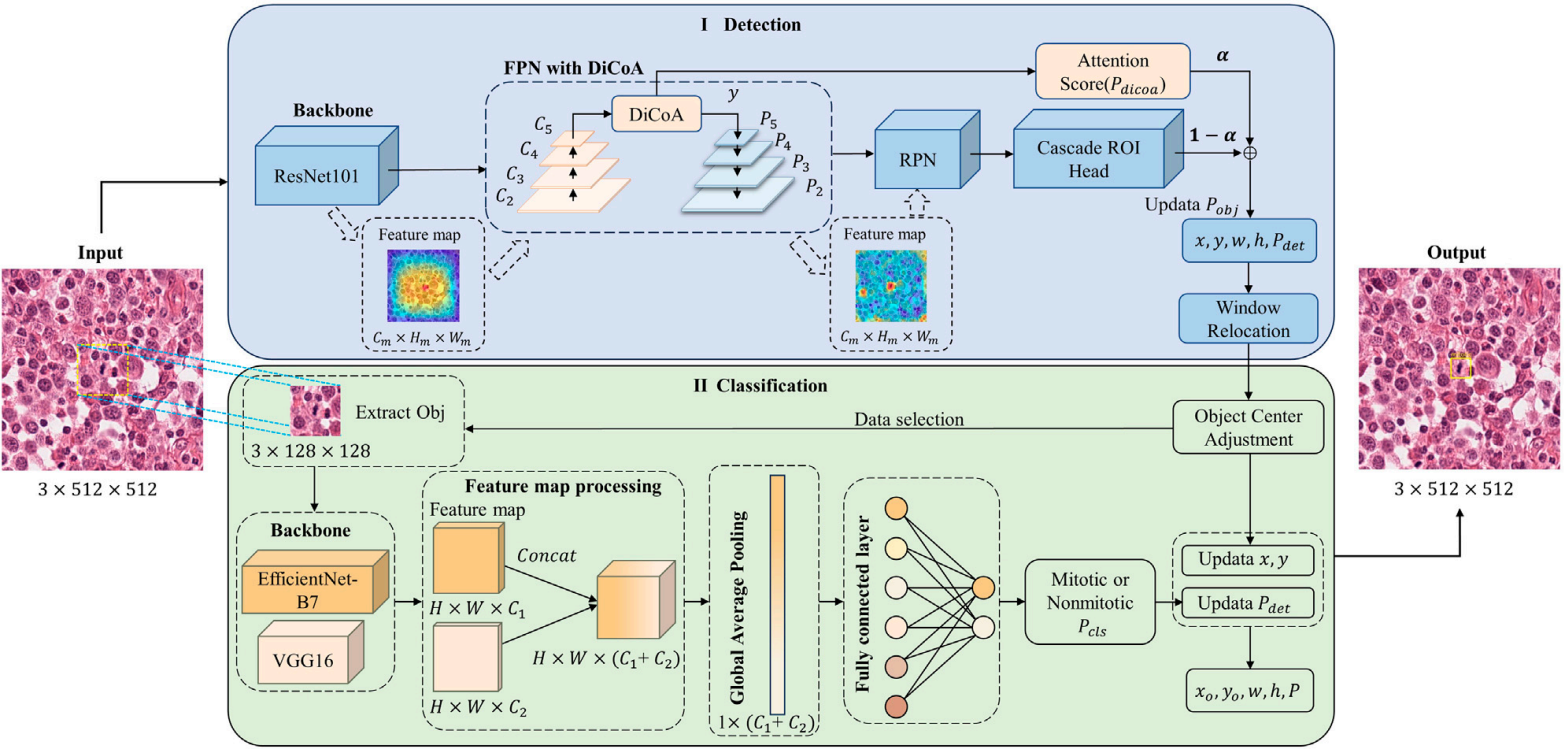
Mitosis detection model overall architecture diagram. I. Detection stage; II. Classification stage. Where $C_{m}$ , $H_{m}$ , $W_{m}$ and represent the channels, height, and width of the feature map, respectively.

Figure 3 shows the structure of our DilCasNet, which comprises two stages: detection and classification. In the detection stage, we employ the Cascade R-CNN object detection network and introduce the DiCoA attention module to predict the positions of mitotic figures in WSI. Subsequently, a window relocation algorithm is applied to reassess low-quality false-positive predictions around the image borders, as illustrated in Figure 3 (I). In the classification stage, we refine the detected targets by center adjustment to better align with the image center. We then incorporate EfficientNet-B7 and VGG16 pre-trained models to reevaluate the confidence scores for each image’s targets, resulting in the final predictions, as depicted in Figure 3 (II).

#### 3.2.3 Detection stage

In the detection stage, we employed the Cascade R-CNN object detection network with an input image size of 3×512×512 . The network outputs a set of bounding boxes { $\left.x,y,w,h,P_{\det}\right\}$ , where $\left(x,y\right)$ represents the center coordinates of the predicted target, w and h denote the width and height of the bounding box, and $P_{\det}$ indicates the confidence of a positive target.

Figure 3 (I) illustrates that our model utilizes ResNet-101 (He et al., 2016) as the backbone network to extract features. These features are fed into a Feature Pyramid Network (FPN) layer enhanced with DiCoA to integrate multi-scale feature information. The DiCoA module is incorporated during the bottom-up process of the FPN module, positioned after the 1×1 convolution layer of the C5 layer. This adjustment allows the feature extraction regions to adapt to the actual size of mitotic cells, as detailed in Supplementary Data SA.3.

After undergoing DiCoA processing, the feature map will yield weighted feature map y and dilated contextual attention scores DPS . The feature map y is input into the Top-down process of the FPN module at the P5 layer to facilitate the transmission of high-level semantic information to lower-level feature maps. Simultaneously, DPS is transformed into a scalar result $P_{dicoa}$ .

The multi-scale feature maps fused by the FPN are input into the Region Proposal Network (RPN) (Ren et al., 2015) for generating candidate regions of mitotic cells. Subsequently, these target candidate regions are passed through the Cascade ROI Head, which consists of a series of cascaded classification heads and regression heads. This process yields the regression parameters x,y,w,h for the bounding box of the target, along with the original target confidence score $P_{obj}$ . Finally, the original target confidence $P_{obj}$ and the attention score $P_{dicoa}$ from DiCoA are weighted by the factor α , resulting in the ultimate confidence $P_{\det}$ or the mitotic cell bounding box. The update is formulated as follows in Eq. 4:

$$P_{\det}=\left(1-\alpha \right)P_{obj}+\alpha P_{dicoa}$$ (4)

In this context, where $\alpha \in \left[0,1\right]$ represents the confidence allocation weight for DiCoA, we set the weight ( α ) to 0.5 (refer to Supplementary Data SB.5).

To maintain model performance stability during data sampling, we opt not to employ dynamic queries. Instead, we sample from the WSI and then train and test. Simultaneously, within the DiCoA module, we set the channel number of the 2D convolution to 256, the kernel size to 1, and the stride to 1. The neighborhood size ( k ) is set to 3 (refer to Supplementary Data SB.2), resulting in D being 9. The dilation value ( δ ) is set to 1. The configuration for $\rho_{D}^{\delta }\left(i,j\right)$ is provided in Supplementary Data SA.2.1.

Following the detection stage, we applied a window relocation method (Piansaddhayanaon et al., 2023) to eliminate low-quality predictions around the borders of the sliding window frames.

The detailed structural parameters of the detection stage can be found in Supplementary Data SA.6.1.

#### 3.2.4 Classification stage

The classification stage occurs after the target detection stage, as illustrated in Figure 3 (II). Initially, we employ the target center adjustment method (Piansaddhayanaon et al., 2023), which adjusts the extracted target center coordinates $\left(x,y\right)$ to the image center $\left(x_{o},y_{o}\right)$ , with specific update details outlined in Supplementary Data SA.4. Subsequently, the EfficientNet-B7 and VGG16 pre-trained models receive input images of size 3×128×128 and generate feature map outputs with consistent width and height, denoted as ${H\times W\times C}_{1}$ and ${H\times W\times C}_{2}$ , respectively. Here, $C_{1}$ and $C_{2}$ represent the channel numbers of the feature maps from different pre-trained models. These two feature maps are concatenated along the channel dimension to form a feature map of size $H\times W\times \left(C_{1}+C_{2}\right)$ . Following this, a 2D global average pooling operation is applied to compress the concatenated feature map to dimensions $1\times \left(C_{1}+C_{2}\right)$ , and then passed through a fully connected layer to output the target confidence. The final output result is utilized to update the results from the detection stage, following the updating method of DeepMitosis (Li et al., 2018). This involves weighting the confidence values $P_{\det}$ from the detection stage and $P_{cls}$ from the classification stage with a weight parameter ω to obtain the ultimate target confidence P , as expressed in formula 5.

$$P=\omega P_{\det}+\left(1-\omega \right)P_{cls}$$ (5)

Where $\omega \in \left[0,1\right]$ is the confidence allocation weight, and Supplementary Data SB.6 explores the setting of this parameter.

The detailed structural parameters of the classification stage can be found in Supplementary Data SA.6.2.

### 3.3 Experimental setup

All experiments in this study were conducted on a computer running the Ubuntu operating system, utilizing the MMDetection (Chen et al., 2019) detection framework, complemented by the Pytorch1.9 and the TensorFlow deep learning library. Our computational setup included an Intel(R) Xeon(R) Silver 4110 CPU @ 2.10 GHz processor and three GeForce RTX 2080 Ti graphics cards.

#### 3.3.1 Detection stage

In the MMDetection object detection framework, we utilized ImageNet (Deng et al., 2010) pre-trained weights to initialize the network’s backbone. We employed the data sampling strategy from Piansaddhayanaon et al. (2023), randomly selecting 5,000 images of size 512×512 from each training WSI for training. During training, we set the batch size to 4 and employed random flips, standard photometric augmentations, and other methods to mitigate the risk of overfitting. Stochastic gradient descent (SGD) was the optimizer. The learning rate followed a stepwise constant decay strategy, starting at $10^{-3}$ . After the fifth and seventh epochs, the learning rate was divided by 10, reaching a final decay to $10^{-5}$ . The maximum number of training epochs was set to 8.

#### 3.3.2 Classification stage

In the classification stage, initially, we adjust the detected positions of mitotic cells (Piansaddhayanaon et al., 2023), and detailed experimental settings can be found in Supplementary Data SA.5. Subsequently, we employ EfficientNet-B7 and VGG16 models pre-trained on ImageNet as the backbone of the network. The input image resolution is set to 128×128 , obtained through active learning data sampling methods (Piansaddhayanaon et al., 2023). We set the batch size to 32 and applied data augmentation techniques, including random translation, random flipping, and standard photometric augmentation. The model is trained using the Adam optimize, with a total of 24,000 training iterations. The initial learning rate is set to $5\times 10^{-4}$ . Dynamic updates were implemented by dividing the learning rate by 10 at the 15,000th and 21,000th iterations, ultimately decaying to $10^{-6}$ .

### 3.4 Model performance evaluation indicators

This paper employs commonly used evaluation metrics, including recall(sensitivity), precision, F1-Score, accuracy, and specificity, as presented in Formulas 6–10. The F1-Score is obtained by calculating the harmonic mean of precision and recall.

$$Recall\left(Sensitivity\right)=\frac{TP}{TP+FN}$$ (6)

$$Precision=\frac{TP}{TP+FP}$$ (7)

$$F1=\frac{2\times Recall\times Precision}{Recall+Precision}$$ (8)

$$Accuracy=\frac{TP+TN}{TP+TN+FP+FN}$$ (9)

$$Specificity=\frac{TN}{TN+FP}$$ (10)

Where TP represents the number of correctly predicted positive samples, FP denotes incorrectly predicted positive samples, FN represents incorrectly predicted negative samples, and TN denotes the number of correctly predicted negative samples.

## 4 Results

### 4.1 Ablation experiments

#### 4.1.1 Model exploration

As shown in Table 1, the performance of the model, combining the detection and classification stages, is superior to that of using only the detection model. In both the integrated model with both detection and classification stages and the case of using only the detection model, the performance of the model is improved with the addition of the DiCoA module.

**TABLE 1:** The ablation experiment comparison between add DiCoA and EfficientNet-B7 + VGG16 on the CMC dataset.

Detector	DiCoA	EfficientNet-B7 + VGG16	Test CMC (%)		
			F1	Precision	Recall
Cascade R-CNN			68.0	69.8	66.3
	✓		74.2	72.8	75.8
		✓ **^a^**	82.2	81.4	83.0
	✓	✓ **^a^**	82.9	82.6	83.2

^a^ Integration of the detection and classification stages.

#### 4.1.2 Detection stage: comparative analysis of different attention modules

As shown in Table 2, on the Cascade R-CNN detection network, our proposed DiCoA attention module, compared to the result without any modifications, exhibited improvements in Recall and F1 by more than 7.5% and 4%, respectively, while experiencing a slight decrease in Precision by 0.1%. In contrast, incorporating the CBAM attention module on the Cascade R-CNN detection network resulted in a 0.5% increase in Recall but led to reductions of 5% and 2% in Precision and F1, respectively. Additionally, for this task, CCNet and SENet attention modules did not yield performance enhancements on the Cascade R-CNN network.

**TABLE 2:** Comparative analysis of various attention modules on cascade R-CNN detection network.

Detector	Method	Test CMC (%)		
		F1	Precision	Recall
Cascade R-CNN	—(Piansaddhayanaon et al., 2023)^a^	70.2	72.9	67.9
	+ SENet (Hu et al., 2020)^b^	67.6	69.4	66.0
	+ CCNet (Huang et al., 2018) ^b^	67.7	67.9	67.6
	+ CBAM (Woo et al., 2018)^b^	68.2	67.9	68.4
	+ DiCoA^c^	74.2	72.8	75.8

^a^ No improvement methods were implemented.

^b^ The attention mechanism proposed in the article.

^c^ The attention module approach proposed by us.

#### 4.1.3 Classification stage: combining pre-trained models for comparison

After the detection stage, we further investigated the impact of combining different pre-trained models on the performance of the classification stage. According to the results in Supplementary Table SB.11, the combination of two pre-trained models, EfficientNet-B7 and VGG16, achieved optimal performance. Compared to using only the VGG16 model, integrating multiple pre-trained models (InPreMo) resulted in improvements of 2.8%, 0.4%, and 1.6% in Precision, Recall, and F1, respectively. Compared to the EfficientNet-B7 model alone, InPreMo exhibited increases of 1.4% in Recall and 0.3% in F1. Furthermore, when compared to the combination of three pre-trained models (EfficientNet-B7, Resnet50, and VGG16), the combination of two pre-trained models (EfficientNet-B7 and VGG16) suggested higher Recall and F1 by 2.3% and 0.3%, respectively, while experiencing a decrease of 1.8% in Precision. Additionally, Supplementary Data SB.9 provides a detailed information on the sensitivity, specificity, and confusion matrix assessments for various classification models.

### 4.2 Comparison with existing literature

As shown in Table 3, our improved method achieved superior results on the CMC dataset compared to existing literature. In contrast to the holistic approach employing the RetinaNet network, our method demonstrates an overall performance improvement of over 5% in Precision, Recall, and F1. Compared to the pipeline of Cascade R-CNN network, our method demonstrates improvements of 0.6%, 1.3%, and 1% in Precision, Recall, and F1, respectively. Additionally, in comparison to the full pipeline of the Faster-RCNN network, our process exhibits an enhancement of 2.4% in Precision, a 0.6% improvement in F1, and a 1.3% decrease in Recall.

**TABLE 3:** Comparison of the proposed method with existing approaches.

Detector	Method	Test CMC (%)			Test CCMCT (%)		
		F1	Precision	Recall	F1	Precision	Recall
RetinaNet (Aubreville et al., 2020)^a^	Detection stage	72.6	69.7	75.8	62.8	57.7	68.8
	Full Pipeline	77.5	77.0	77.9	82.0	82.8	81.2
Faster-RCNN (Piansaddhayanaon et al., 2023)^a^	Detection stage	70.4	71.1	69.7	78.2	78.5	77.9
	Full Pipeline	82.3	80.2	84.5	83.2	83.0	83.4
Cascade R-CNN (Piansaddhayanaon et al., 2023)^a^	Detection stage	70.2	72.9	67.9	75.8	76.5	75.1
	Full Pipeline	81.9	82.0	81.9	82.9	83.2	82.6
Cascade R-CNN	Detection stage^b^	74.2	72.8	75.8	77.4	77.6	77.2
	Full Pipeline^c^	82.9	82.6	83.2	83.0	83.2	82.9

^a^ Results obtained from the article.

^b^ Incorporating our attention module.

^c^ The final results obtained by our approach.

After incorporating the improved DiCoA attention module, compared to the Cascade R-CNN detection network, the detection stage exhibited significant improvements of 7.9% and 4% in Recall and F1, respectively. Relative to the Faster R-CNN and the RetinaNet methods, notable enhancements of 3.8% and 1.6% in F1 were observed. Significance testing using a T-test, presented in Supplementary Data SB.4, indicated *p* values <0.001 for the F1, demonstrating the statistical significance of using the Cascade R-CNN with the added DiCoA attention module over other methods.

Furthermore, we conducted additional evaluation of the model using the CCMCT dataset. Our approach achieved the best performance in Precision compared to the baseline model. Although the F1 score and Recall were slightly lower by 0.2% and 0.5%, respectively, compared to the performance obtained with the Faster-RCNN model, our method still maintained an advantage over other benchmark models.

The above results indicate that our method enhances the detection performance of mitotic cells.

### 4.3 End-to-end evaluation experiment

In an end-to-end setting, following the definition (Meuten et al., 2015) of mitotic cell counting, we determined the region with the highest predicted mitotic cell count (High-Power Field, HPF) (Bertram et al., 2020) by counting mitotic shapes in 10 high-power fields (HPFs) of $2.37\;{mm}^{2}$ each, represented by rectangular windows of size 7110×5333 pixels. Once the HPF region for the WSI was identified, mitotic cell counting was performed either in a fully automated (GA) manner or through a human-machine interactive approach (GB). Under the fully automated setting, the predicted mitotic cell count in the selected HPF was used as the final mitotic cell count. In the human-machine interactive setting, mitotic cell counting was determined based on the annotated mitotic shapes in the selected HPF. Table 4 reports the Mean Absolute Percentage Error (MAPE) and Mean Absolute Error (MAE) at the prediction threshold with the lowest MAPE, indicating a significant improvement in mitotic cell counting on the CMC dataset in a human-machine collaborative environment.

**TABLE 4:** The end-to-end performance of the proposed method, as evaluated on the CMC dataset.

Dataset	Method	GA		GB	
		MAPE	MAE	MAPE	MAE
CMC	ReCasNet (Piansaddhayanaon et al., 2023)^a^	5.6	1.9	5.6	1.6
	Ours	5.8	2.0	4.3	0.9

^a^ Results obtained from the article.

## 5 Discussion

To construct a more accurate model for mitotic cell detection, we devised a two-stage (detection and classification) task model. In the detection stage, we innovatively designed the DiCoA attention module. In the classification stage, we ingeniously proposed a method that integrates multiple pre-trained models to identify mitotic cells. We achieved improved performance on the CMC dataset.

Attention mechanisms are employed to capture crucial features in data, leading to a significant enhancement in model performance (Brauwers and Frasincar, 2022). Despite the diverse types of attention mechanisms proposed, there is limited literature on how to choose an appropriate attention mechanism for mitotic cell identification in cancer. Therefore, we investigated the application of some commonly used attention mechanisms [SENet, which assists the network in automatically capturing the importance of each feature channel (Hu et al., 2020); CCNet, which helps the network capture long-range dependencies between feature pixels (Huang et al., 2018); CBAM, which enhances attention in both spatial and channel dimensions (Woo et al., 2018)] in the task of mitotic cell recognition. As shown in Table 2, while these methods bring varying degrees of performance improvement in their respective domains, their ability to enhance the extraction of advanced features related to mitotic cells is limited. To better extract features of mitotic cells, we proposed a novel DiCoA module to capture remote dependencies between features of mitotic cells. As shown in Table 1, the use of the DiCoA attention module benefits the model in both the single detection stage and the combined detection and classification stages. The introduction of DiCoA reduces false negatives and false positives in mitotic predictions (Supplementary Data SB.7). Simultaneously, as demonstrated in Table 2, the overall performance (Precision, Recall, and F1) with the inclusion of the DiCoA attention module consistently exceeds 72%, while combining CBAM, SENet, and CCNet attention modules yields an overall performance of only 69%.

In previous studies, researches (Irshad et al., 2013; 2014b; Lu and Mandal, 2014; Paul and Mukherjee, 2015; Nateghi et al., 2017) extracted features of mitotic cells manually and subsequently employed machine learning methods for mitotic cell identification. While these methods exhibited remarkable interpretability, they necessitated extensive data preprocessing and feature engineering. In contrast, our approach employs an end-to-end algorithm, leveraging the DiCoA attention mechanism and pre-trained models for enhanced feature extraction and application, thereby improving model performance. With the rise of deep learning, it has been applied in mitotic cell recognition (Cireşan et al., 2013; Zerhouni et al., 2017; Li et al., 2018; Alom et al., 2020). Two fully annotated WSI datasets CCMCT and CMC were introduced, and mitotic cell detection was performed using RetinaNet, followed by classification using ResNet18, achieving a baseline performance. To address the challenge of inconsistent data distribution between detection and classification networks, an improved two-stage framework, ReCasNet (Piansaddhayanaon et al., 2023) was proposed for mitotic detection in CCMCT and CMC datasets. Despite promising results on CCMCT and CMC datasets in existing studies, considering the complexity of mitotic classification detection and model training, the full potential of model performance has yet to be fully explored. As shown in Supplementary Data SB.10, we have summarized and organized various approaches in this field (Cireşan et al., 2013; Albarqouni et al., 2016; Zerhouni et al., 2017; Aubreville et al., 2020; Sebai et al., 2020; Piansaddhayanaon et al., 2023). With an increase in the number of data samples, the performance of single-stage models is limited, and the adoption of two-stage models can further enhance model performance. However, not all two-stage models yield satisfactory results, indicating the need for further exploration. To enhance the model’s performance in mitotic cell recognition and fully exploit the potential of deep learning methods, we developed the DiCoA module, combined with FPN, to identify mitotic cells with diverse scales and shapes. Additionally, we introduced the InPreMo method for fine-grained mitotic classification. As shown in Table 3, compared to the best results of existing research on the CMC dataset (Piansaddhayanaon et al., 2023), our approach achieved an improvement of over 0.5% in Precision and F1. In the detection stage, we introduced the DiCoA module on the Cascade R-CNN network. Compared to the use of Cascade R-CNN and Faster-RCNN networks (Piansaddhayanaon et al., 2023), our approach demonstrated an improvement of over 6% in Recall and over 3.5% in F1. Finally, we evaluated our method in an end-to-end setting. In a human-machine collaborative scenario, our approach, denoted as MC, exhibited a 43.8% reduction in Mean Absolute Error (MAE) (see Table 4).

To enhance the performance in the classification stage, manually extracted features were fused with those obtained from CNN into three classifiers (Wang et al., 2014), achieving improved performance while minimizing computational resource demands. However, manual feature extraction requires domain-specific expertise and often struggles to adapt to large-scale datasets. A deep belief network with multiple classifiers (Beevi et al., 2017) was proposed to segment nuclear regions from clinical images. This approach utilizes multiple classifiers and determines the final outcome through majority voting, resulting in enhanced performance. However, precise nuclear segmentation is required for training effective classifiers, and the training of multiple classification models is complex. To address this, we propose a straightforward multi-pre-trained fusion method, combining two distinct pre-trained models, EfficientNet-B7 and VGG16. Compared to using the VGG16 model alone, our approach suggested improvements of over 1% in Precision and F1. Additionally, relative to the EfficientNet-B7 model, we achieved increases of over 0.2% in Recall and F1. These results indicate that the InPreMo method can effortlessly integrate different pre-trained models, leading to effective performance enhancement.

In the detection stage, we compared the results of multiple detection models (Supplementary Data SB.1) and ultimately selected Cascade R-CNN, which demonstrated the best performance, as our detection model. When utilizing the InPreMo approach, the relationship between the number of stacked models and performance is not linear. As shown in Supplementary Data SB.8, compared to the ensemble of models utilizing EfficientNet-B7, Resnet50, and VGG16 pre-trained methods, combining EfficientNet-B7 and VGG16 pre-trained models yielded a more significant performance improvement while reducing model complexity. We also attempted the advanced CNN classification model ConvNeXt (Liu et al., 2022), but its performance in this task was limited and, therefore, not included. Additionally, constrained by computational resources, we conducted model performance evaluation only on the relatively smaller CMC dataset. Furthermore, despite our method achieving a modest improvement of only 0.5% over the best results from existing research, considering the ubiquity of our approach and the intricate diversity of mitotic cells, our results are deemed acceptable.

It is noteworthy that, when updating the feature map and bounding box confidence of mitotic cells using DiCoA, we found the optimal threshold for mitotic cell bounding boxes to be 0.48 (refer to Supplementary Data SB.3). The reason for this is that, as shown in Eq. 4, we add attention scores from the feature map to the bounding box confidence of the original network, giving it a weight of 0.5. This changes the network’s confidence.

Updating the confidence of mitotic cell bounding boxes with DiCoA may lead to a decrease in box confidence. If adaptation to other domains is required, it may be worth considering not updating the confidence of the targets. Additionally, the InPreMo method necessitates the selection of an appropriate model depending on the specific task, which warrants further exploration in other studies. Moreover, in terms of model complexity, the introduction of both DiCoA and InPreMo tends to increase the model’s complexity to some extent. Although our enhancements have improved the model’s capability to extract features related to mitotic cells, further research and optimization are still required to enhance the performance of the network in mitotic cell recognition. Furthermore, in upcoming research, we will further consider addressing variability both between and within observers to ensure the accuracy and reliability of the data.

## 6 Conclusion

We developed the DilCasNet model for more accurate identification of mitotic cells by introducing two key improvements to the two-stage mitotic cell detection method. Firstly, we proposed the DiCoA module with sparse global attention, effectively enhancing the detection network’s ability to capture long-range dependencies between features of mitotic cells. This enables the model to better recognize mitotic cells of varying sizes and shapes, reducing false-negative and false-positive predictions while significantly improving overall performance. Secondly, we ingeniously integrated the EfficientNet-B7 and VGG16 pre-trained models, enhancing the model’s performance in the classification stage. This approach provides a novel choice for current classification networks. Our method demonstrated improved performance in detecting mitotic cells on the CMC dataset.

## Data Availability

The original contributions presented in the study are included in the article/Supplementary Material, further inquiries can be directed to the corresponding author.

## References

[B1] Albarqouni S. Baur C. Achilles F. Belagiannis V. Demirci S. Navab N. (2016). AggNet: deep learning from crowds for mitosis detection in breast cancer histology images. IEEE Trans. Med. Imaging 35, 1313–1321. 10.1109/TMI.2016.2528120 26891484

[B2] Alom M. Z. Aspiras T. Taha T. M. Bowen T. Asari V. K. (2020). MitosisNet: end-to-end mitotic cell detection by multi-task learning. IEEE Access 8, 68695–68710. 10.1109/ACCESS.2020.2983995

[B3] Aubreville M. Bertram C. A. Donovan T. A. Marzahl C. Maier A. Klopfleisch R. (2020). A completely annotated whole slide image dataset of canine breast cancer to aid human breast cancer research. Sci. Data 71 (7), 417–510. 10.1038/s41597-020-00756-z PMC7699627 33247116

[B4] Beevi K. S. Nair M. S. Bindu G. R. (2017). A multi-classifier system for automatic mitosis detection in breast histopathology images using deep belief networks. IEEE J. Transl. Eng. Heal. Med. 5, 4300211. 10.1109/JTEHM.2017.2694004 PMC5480254 29018640

[B5] Bertram C. A. Aubreville M. Gurtner C. Bartel A. Corner S. M. Dettwiler M. (2020). Computerized calculation of mitotic count distribution in canine cutaneous mast cell tumor sections: mitotic count is area dependent. Vet. Pathol. 57, 214–226. 10.1177/0300985819890686 31808382

[B6] Bertram C. A. Aubreville M. Marzahl C. Maier A. Klopfleisch R. (2019). A large-scale dataset for mitotic figure assessment on whole slide images of canine cutaneous mast cell tumor. Sci. Data 61 (6), 274–279. 10.1038/s41597-019-0290-4 PMC6872565 31754105

[B7] Brauwers G. Frasincar F. (2022). A general survey on attention mechanisms in deep learning. IEEE Trans. Knowl. Data Eng. 35, 3279–3298. 10.1109/TKDE.2021.3126456

[B8] Cai Z. Vasconcelos N. (2017). Cascade R-CNN: delving into high quality object detection. Proc. IEEE Comput. Soc. Conf. Comput. Vis. Pattern Recognit., 6154–6162. 10.1109/CVPR.2018.00644

[B9] Chen K. Wang J. Pang J. Cao Y. Xiong Y. Li X. (2019). MMDetection: open MMLab detection toolbox and benchmark. https://arxiv.org/abs/1906.07155.

[B10] Cireşan D. C. Giusti A. Gambardella L. M. Schmidhuber J. (2013). Mitosis detection in breast cancer histology images with deep neural networks. Med. Image Comput. Comput. Assist. Interv. 16, 411–418. 10.1007/978-3-642-40763-5_51 24579167

[B11] Cree I. A. Tan P. H. Travis W. D. Wesseling P. Yagi Y. White V. A. (2021). Counting mitoses: SI(ze) matters. Mod. Pathol. 349 (34), 1651–1657. 10.1038/s41379-021-00825-7 PMC8376633 34079071

[B12] Dai L. J. Ma D. Xu Y. Z. Li M. Li Y. W. Xiao Y. (2023). Molecular features and clinical implications of the heterogeneity in Chinese patients with HER2-low breast cancer. Nat. Commun. 141 (14), 5112–5113. 10.1038/s41467-023-40715-x PMC10444861 37607916

[B13] Deng J. Dong W. Socher R. Li L.-J. Li K. Fei-Fei L. (2010). “ImageNet: a large-scale hierarchical image database,” in 2009 IEEE Conference on Computer Vision and Pattern Recognition, Miami, FL, USA, June, 2010, 248–255. 10.1109/CVPR.2009.5206848

[B14] Gurcan M. N. Boucheron L. E. Can A. Madabhushi A. Rajpoot N. M. Yener B. (2009). Histopathological image analysis: a review. IEEE Rev. Biomed. Eng. 2, 147–171. 10.1109/RBME.2009.2034865 20671804 PMC2910932

[B15] Hassani A. Shi H. (2022). Dilated neighborhood attention transformer. https://arxiv.org/abs/2209.15001.

[B16] He K. Zhang X. Ren S. Sun J. (2016). “Deep residual learning for image recognition,” in Proc. IEEE Comput. Soc. Conf. Comput. Vis. Pattern Recognit, Las Vegas, NV, USA, June, 2016, 770–778. 10.1109/CVPR.2016.90

[B17] Hu J. Shen L. Albanie S. Sun G. Wu E. (2020). Squeeze-and-Excitation networks. IEEE Trans. Pattern Anal. Mach. Intell. 42, 2011–2023. 10.1109/TPAMI.2019.2913372 31034408

[B18] Huang G. Liu Z. Van Der Maaten L. Weinberger K. Q. (2017). “Densely connected convolutional networks,” in Proc. - 30th IEEE Conf. Comput. Vis. Pattern Recognition, CVPR 2017, Honolulu, Hawaii, January, 2017, 2261–2269. 10.1109/CVPR.2017.243

[B19] Huang Z. Wang X. Wei Y. Huang L. Shi H. Liu W. (2018). CCNet: criss-cross attention for semantic segmentation. IEEE Trans. Pattern Anal. Mach. Intell. 45, 6896–6908. 10.1109/TPAMI.2020.3007032 32750802

[B20] Ibrahim A. Lashen A. Toss M. Mihai R. Rakha E. (2022). Assessment of mitotic activity in breast cancer: revisited in the digital pathology era. J. Clin. Pathol. 75, 365–372. 10.1136/JCLINPATH-2021-207742 34556501

[B21] Ibrahim A. Lashen A. G. Katayama A. Mihai R. Ball G. Toss M. S. (2021). Defining the area of mitoses counting in invasive breast cancer using whole slide image. Mod. Pathol. 356 (35), 739–748. 10.1038/s41379-021-00981-w PMC9174050 34897279

[B22] Irshad H. Gouaillard A. Roux L. Racoceanu D. (2014a). Multispectral band selection and spatial characterization: application to mitosis detection in breast cancer histopathology. Comput. Med. Imaging Graph. 38, 390–402. 10.1016/J.COMPMEDIMAG.2014.04.003 24831181

[B23] Irshad H. Gouaillard A. Roux L. Racoceanu D. (2014b). “Spectral band selection for mitosis detection in histopathology,” in 2014 IEEE 11th Int. Symp. Biomed. Imaging, ISBI, Beijing, China, April, 2014, 1279–1282. 10.1109/ISBI.2014.6868110

[B24] Irshad H. Jalali S. Roux L. Racoceanu D. Hwee L. J. Naour G.Le (2013). Automated mitosis detection using texture, SIFT features and HMAX biologically inspired approach. J. Pathol. Inf. 4, 12. 10.4103/2153-3539.109870 PMC3678748 23766934

[B25] Lecun Y. Bengio Y. Hinton G. (2015). Deep learning. Nat 521, 436–444. 10.1038/nature14539 26017442

[B26] Li C. Wang X. Liu W. Latecki L. J. (2018). DeepMitosis: mitosis detection via deep detection, verification and segmentation networks. Med. Image Anal. 45, 121–133. 10.1016/J.MEDIA.2017.12.002 29455111

[B27] Lin T.-Y. Dollár P. Girshick R. He K. Hariharan B. Belongie S. (2016). “Feature Pyramid networks for object detection,” in 2016 IEEE Conference on Computer Vision and Pattern Recognition (CVPR), Honolulu, HI, USA, July, 2016. 10.48550/arxiv.1612.03144

[B28] Liu Z. Mao H. Wu C. Y. Feichtenhofer C. Darrell T. Xie S. (2022). “A ConvNet for the 2020s,” in Proc. IEEE Comput. Soc. Conf. Comput. Vis. Pattern Recognit, New Orleans, LA, USA, June, 2022, 11966–11976. 10.1109/CVPR52688.2022.01167

[B29] Lu C. Mandal M. (2014). Toward automatic mitotic cell detection and segmentation in multispectral histopathological images. IEEE J. Biomed. Heal. Inf. 18, 594–605. 10.1109/JBHI.2013.2277837 24608059

[B30] Ludovic R. Daniel R. Nicolas L. Maria K. Humayun I. Jacques K. (2013). Mitosis detection in breast cancer histological images an ICPR 2012 contest. J. Pathol. Inf. 4, 8. 10.4103/2153-3539.112693 PMC3709417 23858383

[B31] Mahmood T. Arsalan M. Owais M. Lee M. B. Park K. R. (2020). Artificial intelligence-based mitosis detection in breast cancer histopathology images using faster R-CNN and deep CNNs. J. Clin. Med. 9, 749. 10.3390/JCM9030749 32164298 PMC7141212

[B32] Mathew T. Kini J. R. Rajan J. (2021). Computational methods for automated mitosis detection in histopathology images: a review. Biocybern. Biomed. Eng. 41, 64–82. 10.1016/J.BBE.2020.11.005

[B33] Meuten D. J. Moore F. M. George J. W. (2015). Mitotic count and the field of view area: time to standardize. Vet. Pathol. 53, 7–9. 10.1177/0300985815593349 26712813

[B34] MITOS-ATYPIA-14 (2014). Mitos-Atypia-14-Dataset. Available at: https://mitos-atypia-14.grand-challenge.org/Dataset/.

[B35] Nateghi R. Danyali H. Helfroush M. S. (2017). Maximized inter-class weighted mean for fast and accurate mitosis cells detection in breast cancer histopathology images. J. Med. Syst. 41, 146–215. 10.1007/s10916-017-0773-9 28808813

[B36] Pan S. J. Yang Q. (2010). A survey on transfer learning. IEEE Trans. Knowl. Data Eng. 22, 1345–1359. 10.1109/TKDE.2009.191

[B37] Paul A. Mukherjee D. P. (2015). Mitosis detection for invasive breast cancer grading in histopathological images. IEEE Trans. Image Process. 24, 4041–4054. 10.1109/TIP.2015.2460455 26219094

[B38] Piansaddhayanaon C. Santisukwongchote S. Shuangshoti S. Tao Q. Sriswasdi S. Chuangsuwanich E. (2023). ReCasNet: improving consistency within the two-stage mitosis detection framework. Artif. Intell. Med. 135, 102462. 10.1016/J.ARTMED.2022.102462 36628784

[B39] Ren S. He K. Girshick R. Sun J. (2015). Faster R-CNN: towards real-time object detection with region proposal networks. IEEE Trans. Pattern Anal. Mach. Intell. 39, 1137–1149. 10.1109/TPAMI.2016.2577031 27295650

[B40] Sebai M. Wang T. Al-Fadhli S. A. (2020). PartMitosis: a partially supervised deep learning framework for mitosis detection in breast cancer histopathology images. IEEE Access 8, 45133–45147. 10.1109/ACCESS.2020.2978754

[B41] Simonyan K. Zisserman A. (2015). “Very deep convolutional networks for large-scale image recognition,” in 3rd Int. Conf. Learn. Represent. ICLR 2015 - Conf. Track Proc., San Diego, CA, USA, May, 2015.

[B42] Tan M. Le Q. V. (2019). “EfficientNet: rethinking model scaling for convolutional neural networks,” in 36th International Conference on Machine Learning, Long Beach, CA, USA, June, 2019, 6105–6114.

[B43] Tran K. A. Kondrashova O. Bradley A. Williams E. D. Pearson J. V. Waddell N. (2021). Deep learning in cancer diagnosis, prognosis and treatment selection. Genome Med. 13, 152. 10.1186/S13073-021-00968-X 34579788 PMC8477474

[B44] Udousoro I. C. (2020). Machine learning: a review. Semicond. Sci. Inf. Devices 2, 5–14. 10.30564/SSID.V2I2.1931

[B45] Veta M. Heng Y. J. Stathonikos N. Bejnordi B. E. Beca F. Wollmann T. (2019). Predicting breast tumor proliferation from whole-slide images: the TUPAC16 challenge. Med. Image Anal. 54, 111–121. 10.1016/J.MEDIA.2019.02.012 30861443

[B46] Veta M. van Diest P. J. Willems S. M. Wang H. Madabhushi A. Cruz-Roa A. (2015). Assessment of algorithms for mitosis detection in breast cancer histopathology images. Med. Image Anal. 20, 237–248. 10.1016/J.MEDIA.2014.11.010 25547073

[B47] Wang H. Cruz-Roa A. Basavanhally A. Gilmore H. Shih N. Feldman M. (2014). Cascaded ensemble of convolutional neural networks and handcrafted features for mitosis detection. Med. Imaging 2014 Digit. Pathol. 9041, 90410B. 10.1117/12.2043902 PMC4479031 26158062

[B48] Wei Z. Cheng S. Cai J. Zeng S. Liu X. Wang Z. (2022). 3D soma detection in large-scale whole brain images via a two-stage neural network. IEEE Trans. Med. Imaging 42, 148–157. 10.1109/tmi.2022.3206605 36103445

[B49] Weng W. Zhu X. (2015). INet: convolutional networks for biomedical image segmentation. IEEE Access 9, 16591–16603. 10.1109/access.2021.3053408

[B50] Woo S. Park J. Lee J. Y. Kweon I. S. (2018). “CBAM: convolutional Block attention module,” in Proc. Eur. Conf. Comput. Vis. 11211 LNCS, Munich, Germany, September, 2018, 3–19. 10.1007/978-3-030-01234-2_1

[B51] Xu Y. Gong M. Wang Y. Yang Y. Liu S. Zeng Q. (2023). Global trends and forecasts of breast cancer incidence and deaths. Sci. Data 10, 334–410. 10.1038/s41597-023-02253-5 37244901 PMC10224917

[B52] Zerhouni E. Lanyi D. Viana M. Gabrani M. (2017). Wide residual networks for mitosis detection. Proc. - Int. Symp. Biomed. Imaging, 924–928. 10.1109/ISBI.2017.7950667

