# Supplementary material for: A novel dilated contextual attention module for breast cancer mitosis cell detection
Source: Front Physiol. 2024 Jan 25;15:1337554. doi: 10.3389/fphys.2024.1337554 (PMC10850563; doi:10.3389/fphys.2024.1337554)
Supplement: Supplementary file 1 [file DataSheet1.pdf]

Supplementary Material

Supplementary A. Additional structure details

A.1. Dataset

A.1.1. CMC Dataset

The CODAEL labels of the CMC dataset are divided into two categories: Mitosis, and Nonmitosis, comprising a total of 13,907 annotated mitosis and 36,379 annotated non-mitosis, as shown in Table A.1. The training set consists of 10,495 annotated mitosis and 27,456 annotated non-mitosis, while the test set includes 3,412 annotated mitosis and 8,923 annotated non-mitosis.

Supplementary Table A.1. Individual WSI Overview within the CMC Dataset under the CODAEL Label.

Case No.	File name	No. of mitotic figures (CODAEL)	No. of non-mitotic cells (CODAEL)	set
**1**	4eee7b944ad5e46c60ce.svs	64	193	test
**2**	a8773be388e12df89edd.svs	74	588	train
**3**	deb768e5efb9d1dcbc13.svs	84	484	train
**4**	e09512d530d933e436d5.svs	102	738	test
**5**	72c93e042d0171a61012.svs	140	691	train
**6**	2d56d1902ca533a5b509.svs	153	367	test
**7**	084383c18b9060880e82.svs	160	560	train
**8**	da18e7b9846e9d38034c.svs	211	1,353	train
**9**	13528f1921d4f1f15511.svs	292	1,136	test
**10**	d0423ef9a648bb66a763.svs	354	1,653	train
**11**	69a02453620ade0edefd.svs	612	1,535	test
**12**	d37ab62158945f22deed.svs	674	1,702	train
**13**	d7a8af121d7d4f3fbf01.svs	720	2,399	train
**14**	460906c0b1fe17ea5354.svs	754	2,468	train
**15**	b1bdee8e5e3372174619.svs	869	1,824	test
**16**	c4b95da36e32993289cb.svs	1,085	2,973	train
**17**	022857018aa597374b6c.svs	1,320	3,130	test
**18**	50cf88e9a33df0c0c8f9.svs	1,337	2,552	train
**19**	3d3d04eca056556b0b26.svs	1,447	2,951	train
**20**	2191a7aa287ce1d5dbc0.svs	1,462	2,632	train
**21**	fa4959e484beec77543b.svs	1,993	4,450	train
	total train	10,495	27,456	
	total test	3,412	8,923	
	total	13,907	36,379	

A.1.1. CCMCT Dataset

Supplementary Table A.2. Individual WSI Overview within the CCMCT Dataset under the ODAEL Label.

Case No.	Slide name	No. of mitotic figures	No. of mitotic figure look-alikes	No. of granulocytes	No. of normal tumor cells set	Set
**1**	2f2591b840e83a4b4358.svs	3	48	2213	1149	train
**2**	ce949341ba99845813ac.svs	4	30	35	1200	train
**3**	91a8e57ea1f9cb0aeb63.svs	6	16	573	1916	train
**4**	9374efe6ac06388cc877.svs	7	17	1531	1567	train
**5**	0e56fd11a762be0983f0.svs	8	262	239	1620	train
**6**	dd6dd0d54b81ebc59c77.svs	11	57	1230	1830	train
**7**	be10fa37ad6e88e1f406.svs	12	55	124	1354	test
**8**	2e611073cff18d503cea.svs	18	137	2556	1136	train
**9**	066c94c4c161224077a9.svs	19	54	1742	1035	train
**10**	285f74bb6be025a676b6.svs	19	48	2895	1837	train
**11**	f3741e764d39ccc4d114.svs	37	115	724	1932	test
**12**	c86cd41f96331adf3856.svs	56	75	2412	1593	test
**13**	2efb541724b5c017c503.svs	66	24	645	621	train
**14**	70ed18cd5f806cf396f0.svs	85	880	1913	578	train
**15**	552c51bfb88fd3e65ffe.svs	119	670	1688	2074	test
**16**	3f2e034c75840cb901e6.svs	571	350	1434	1913	train
**17**	8c9f9618fcaca747b7c3.svs	715	1212	28	3077	test
**18**	c91a842257ed2add5134.svs	759	690	2327	1719	test
**19**	dd4246ab756f6479c841.svs	777	525	2703	2986	test
**20**	8bebdd1f04140ed89426.svs	1000	534	1563	2196	train
**21**	2f17d43b3f9e7dacf24c.svs	1157	477	2719	1625	train
**22**	a0c8b612fe0655eab3ce.svs	1279	1407	2118	1556	train
**23**	ac1168b2c893d2acad38.svs	1329	474	613	4354	train
**24**	fff27b79894fe0157b08.svs	1744	1466	5774	2279	train
**25**	34eb28ce68c1106b2bac.svs	2279	1297	2054	1540	train
**26**	f26e9fcef24609b988be.svs	2380	459	2447	1807	test
**27**	96274538c93980aad8d6.svs	3068	3762	1170	4297	test
**28**	add0a9bbc53d1d9bac4c.svs	3569	1759	415	2198	test
**29**	39ecf7f94ed96824405d.svs	3689	3412	1572	1931	train
**30**	20c0753af38303691b27.svs	4343	2024	1772	3835	train
**31**	c3eb4b8382b470dd63a9.svs	4767	1326	140	9461	train
**32**	1018715d369dd0df2fc0.svs	10984	4303	2070	3135	test
	total train	22404	14340	35331	45179	
	total test	22476	13625	16108	26172	
	total	44880	27965	51439	71351	

The ODAEL labels in the CCMCT dataset consist of four categories: Mitosis, Mitosislike, Granulocyte, and Tumorcell, totaling 44,880 annotated mitosis, 27,965 annotated mitosis-like, 51,439 annotated granulocyte, and 71,351 annotated tumor cells, as detailed in Table A.2. The training set includes 22,404 annotated mitosis, 14,340 annotated mitosis-like, 35,331 annotated granular cells, and 45,179

annotated tumor cells. Meanwhile, the test set comprises 22,476 annotated mitosis, 13,625 annotated mitosis-like, 16,108 annotated granular cells, and 26,172 annotated tumor cells.

A.2. Internal Parameter Configuration of DiCoA

A.2.1 Parameter Configuration

We set $\delta = 1$ and $k = 3, D = 9$. Thus $\rho_D^{\delta}(i,j) \in N^{D\times 2}$ is selected from the following index set: $[(i-2,j-2),(i-2,j),(i-2,j+1),(i,j-2),(i,j),(i,j+2),(i+1,j-2),(i+2,j),(i+2,j+2)]$. At this point, the receptive field (RF) is 5, representing a region of size 5×5, and RF can be calculated using formula (A.1).

$$RF = k + (k-1)(\delta + 1) \quad \text{(A.1)}$$

A.2.2 DiCoA Attention Scores

Firstly, extract the dilated contextual attention score matrix, denoted as $\boldsymbol{DPS}_{D,i,j}^{\delta}$, for a given input feature map $\boldsymbol{x} \in R^{C\times H\times W}$. Transforming it into a scalar result is represented by P_{dicoa}:

$$P_{dicoa} = \frac{1}{(D-1)b_w b_h} \sum_{i=b_x-\frac{b_w}{2}}^{b_x+\frac{b_w}{2}} \sum_{j=b_y-\frac{b_h}{2}}^{b_y+\frac{b_h}{2}} \sum_{n}^{1\sim\frac{D-1}{2},\frac{D+3}{2}\sim D} \boldsymbol{DPS}_{n,i,j}^{\delta} \quad \text{(A.2)}$$

Where (b_x, b_y) represents the coordinate center of the target box, and b_w, b_h denote the width and height of the bounding box, respectively. D represents the total number of pixels extracted in a neighborhood of size k, and δ represents the dilation value.

During the training stage, the attention scores of DiCoA are updated by optimizing the objective loss with respect to the target using the cross-entropy method：

$$L_{DiCoA} = log\left(NLLLoss\left([P_{nondicoa}P_{dicoa}], P_{gt}\right)\right) \quad \text{(A.3)}$$

$$P_{nondicoa} = \frac{1}{b_w b_h} \sum_{i=b_x-\frac{b_w}{2}}^{b_x+\frac{b_w}{2}} \sum_{j=b_y-\frac{b_h}{2}}^{b_y+\frac{b_h}{2}} \boldsymbol{DPS}_{\frac{D+1}{2},i,j}^{\delta} \quad \text{(A.4)}$$

Where P_{gt} represents the ground truth of a bounding box. $NLLLoss$ denotes the negative log-likelihood loss function. $NLLLoss$, log, and $softmax$ together constitute the cross-entropy loss.

A.3. DiCoA Attention Placement Locations

To effectively detect mitotic cells of various shapes, capture long-range dependencies in mitotic cell features, and adapt the extracted feature vectors to the physical sizes of different mitotic cells, we designed the integration of the DiCoA attention module with a multi-scale network. We chose to incorporate the DiCoA attention module in the fifth stage of the Bottom-up process of the FPN, specifically after the output of the 1×1 convolutional layer with a downsampling rate of $2^5 = 32$, denoted as C5. Subsequently, we obtained P2, P3, P4, and P5 through four 1×1 convolution operations, derived from C2, C3, C4, and C5, respectively (as illustrated in Figure A.1). These operations reduced the channel dimensions of the feature maps to C=256, facilitating better processing of features across different scales.

The rationale for incorporating the DiCoA attention module at the C5 stage of the FPN module is as follows: In this study, the image resolution is 0.25 μm/pixel, and a C5 stage feature vector represents a region of length 24 μm, as illustrated in Figure A.1 (a). Figure A.1 (b) demonstrates the annotated size of a mitotic cell with a diameter of 25 μm. The downsampling rate of the C5 stage precisely encompasses the entire region of a mitotic cell. Therefore, by applying DiCoA at the C5 stage, we can better capture information related to mitotic cells. In earlier stages, such as the C4 stage with a downsampling rate of 2^4 or the C3 stage with a downsampling rate of 2^3, utilizing DiCoA cannot adequately capture the information of mitotic cells.

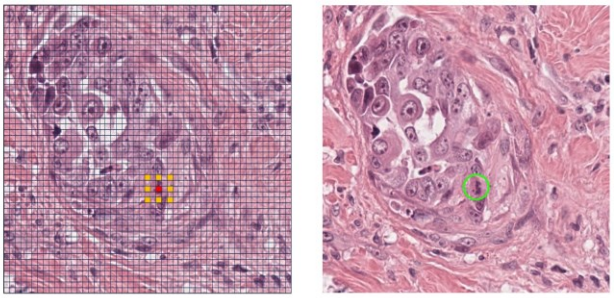

(a) (b)

Supplementary Figure A.1. The schematic representation of the attention receptive field size of DiCoA. (a) Explanation of the attention span for a single pixel, (b) A green circle highlights a mitotic cell.

A.4. Target Center Adjustment

Despite window relocation having reassessed numerous false-positive samples at the boundaries of sliding windows, low-quality bounding boxes persist in the classification stage. We employed the target center adjustment method to mitigate input translation variance. Optimization was achieved by balancing the combination of regression loss L_{reg} and classification loss L_{cls} through the loss function L_{rel}, as expressed in Equation (A.5). Here, the parameter λ_{reg} is utilized to control the weighting of the two losses.

$$L_{rel} = \lambda_{reg} L_{reg} + \left(1 - \lambda_{reg}\right) L_{cls} \quad \text{(A.5)}$$

Where $\lambda_{reg} \in [0,1]$ is the loss allocation weight, we set λ_{reg} to 0.95. The classification loss L_{cls} is computed based on the standard cross-entropy loss between the predicted and true target categories. The regression loss L_{reg} is derived from the calculation of the distance between the predicted and true target centers.

A.5. Experimental Setup for Target Center Adjustment

We employed the target center adjustment method, utilizing DenseNet201 as the network backbone during training. The input image resolution was set to 128×128. The network backbone was initialized with ImageNet pre-trained weights. Training was performed with a batch size of 64 using the Adam optimizer. The initial learning rate for the model was set to 10^{-4}, and during training, it was adjusted to $1/10$ of the initial value after the 22,500th and 27,000th iterations. In each experiment, λ_{reg} was set to 0.95. To enhance the diversity of training data, we applied random flipping and standard

photometric data augmentation techniques during training. For the CMC dataset, the threshold for positive class was set to 0.2.

A.6. Model Architectures

A.6.1 Model Architectures for the Detection Stage

Supplementary Table A.3. Architectures for the Detection Stage.

Detection Stage	Layer Name	Input Size	Output Size	Block
**ResNet101**	Conv1	$512 \times 512 \times 3$	$256 \times 256 \times 64$	Conv 7 × 7, 64, stride 2, padding 3
	Max Pooling	$256 \times 256 \times 64$	$128 \times 128 \times 64$	Conv 3 × 3 max pool, stride 2
	Layer1	$128 \times 128 \times 64$	$128 \times 128 \times 256$	$\begin{bmatrix} 1 \times 1, 128 \\ 3 \times 3, 128 \\ 1 \times 1, 256 \end{bmatrix} \times 3$
	Layer2	$128 \times 128 \times 256$	$64 \times 64 \times 512$	$\begin{bmatrix} 1 \times 1, 256 \\ 3 \times 3, 256 \\ 1 \times 1,512 \end{bmatrix} \times 4$
	Layer3	$64 \times 64 \times 512$	$32 \times 32 \times 1024$	$\begin{bmatrix} 1 \times 1, 512 \\ 3 \times 3, 512 \\ 1 \times 1,1024 \end{bmatrix} \times 23$
	Layer4	$32 \times 32 \times 1024$	$16 \times 16 \times 2048$	$\begin{bmatrix} 1 \times 1, 1024 \\ 3 \times 3, 1024 \\ 1 \times 1,2048 \end{bmatrix} \times 3$
**FPN(Bottom-up)**	C2	$128 \times 128 \times 256$	$128 \times 128 \times 256$	Conv 1 × 1, 256, stride 1
	C3	$64 \times 64 \times 512$	$64 \times 64 \times 256$	Conv 1 × 1, 256, stride 1
	C4	$32 \times 32 \times 1024$	$32 \times 32 \times 256$	Conv 1 × 1, 256, stride 1
	C5	$16 \times 16 \times 2048$	$16 \times 16 \times 256$	Conv 1 × 1, 256, stride 1
**DiCoA**	Q	$16 \times 16 \times 256$	$16 \times 16 \times 256$	Conv 1 × 1, 256, stride 1
	K	$16 \times 16 \times 256$	$16 \times 16 \times 256$	Conv 1 × 1, 256, stride 1
	V	$16 \times 16 \times 256$	$16 \times 16 \times 256$	Conv 1 × 1, 256, stride 1
**FPN(Top-Down)**	P5	$16 \times 16 \times 256$	$16 \times 16 \times 256$	Conv 3 × 3, 256, stride 1, padding 1
	P4	$32 \times 32 \times 256$	$32 \times 32 \times 256$	Conv 3 × 3, 256, stride 1, padding 1
	P3	$64 \times 64 \times 256$	$64 \times 64 \times 256$	Conv 3 × 3, 256, stride 1, padding 1
	P2	$128 \times 128 \times 256$	$128 \times 128 \times 256$	Conv 3 × 3, 256, stride 1, padding 1
**RPN Head**	RPN Conv	$128 \times 128 \times 256$	$64 \times 64 \times 256$	Conv 3 × 3, 256, stride 1, padding 1
	RPN cls	$64 \times 64 \times 256$	$64 \times 64 \times 3$	Conv 1 × 1, 3, stride 1
	RPN reg	$64 \times 64 \times 256$	$64 \times 64 \times 12$	Conv 1 × 1, 12, stride 1
**Cascade RoI Align**	Bbox RoI Extractor	$64 \times 64 \times 256$	$7 \times 7 \times 256$	RoI Align
**Cascade Head**	Shared Fcs	12544	1024	Linear(12544, 1024)
	FC cls	1024	2	Linear(1024, 2)
	FC reg	1024	4	Linear(1024, 4)

A.6.2 Model Architectures for the Detection Stage

Supplementary Table A.4. Architectures for the Classification Stage.

Classification Stage	Layer Name	Input Size	Output Size	Block
**EfficientNet-B7**	Stem	$128 \times 128 \times 3$	$64 \times 64 \times 64$	Conv 3×3
	MBConv1	$64 \times 64 \times 64$	$64 \times 64 \times 192$	Conv $1 \times 1, 3 \times 3$
	MBConv2	$64 \times 64 \times 192$	$32 \times 32 \times 288$	Conv $1 \times 1, 3 \times 3$
	MBConv3	$32 \times 32 \times 288$	$16 \times 16 \times 480$	Conv $1 \times 1, 3 \times 3$
	MBConv4	$16 \times 16 \times 480$	$8 \times 8 \times 960$	Conv $1 \times 1, 3 \times 3$
	MBConv5	$8 \times 8 \times 960$	$8 \times 8 \times 1344$	Conv $1 \times 1, 3 \times 3$
	MBConv6	$8 \times 8 \times 1344$	$4 \times 4 \times 2304$	Conv $1 \times 1, 3 \times 3$
	MBConv7	$4 \times 4 \times 2304$	$4 \times 4 \times 2560$	Conv $1 \times 1, 3 \times 3$
**VGG16**	Conv2D (block1)	$128 \times 128 \times 3$	$128 \times 128 \times 64$	Conv 3×3
	MaxPooling2D	$64 \times 64 \times 64$	$64 \times 64 \times 64$	Pooling 2×2
	Conv2D (block2)	$64 \times 64 \times 64$	$64 \times 64 \times 64$	Conv 3×3
	MaxPooling2D	$64 \times 64 \times 64$	$64 \times 64 \times 128$	Pooling 2×2
	Conv2D (block3)	$64 \times 64 \times 128$	$32 \times 32 \times 128$	Conv 3×3
	MaxPooling2D	$32 \times 32 \times 128$	$16 \times 16 \times 256$	Pooling 2×2
	Conv2D (block4)	$16 \times 16 \times 256$	$16 \times 16 \times 512$	Conv 3×3
	MaxPooling2D	$16 \times 16 \times 512$	$8 \times 8 \times 512$	Pooling 2×2
	Conv2D (block5)	$8 \times 8 \times 512$	$8 \times 8 \times 512$	Conv 3×3
	MaxPooling2D	$8 \times 8 \times 512$	$4 \times 4 \times 512$	Pooling 2×2
**Feature Map Processing**	Concat	$4 \times 4 \times 2560$<br>$4 \times 4 \times 512$	$4 \times 4 \times 3072$	Concat
**Global Average Polling**	Pooling2d	$4 \times 4 \times 3072$	3072	Average pooling
**Full Connected Layer**	Dense	3072	2	Linear(3072, 2)

Supplementary B. Additional ablation studies

B.1. Detection Model Experiments

Supplementary Table B.5. Experimental Results of Different Models.

Detector	Test CMC（%）		
	F1	Precision	Recall
**Cascade R-CNN**	68.0	70.0	66.3
**Faster-RCNN**	65.9	67.7	64.2
**YOLOF**	61.3	64.1	58.7
**RetinaNet**	57.0	63.0	52.1

Table B.5 displays the experimental results of different detection models tested on the CMC dataset, with the outcome indicating the superior performance of the Cascade R-CNN detection network.

B.2. Comparison of Internal Parameter Structural Adjustments in DiCoA

Supplementary Table B.6. Experiment on Internal Parameter Structural Adjustment of DiCoA.

Detector	Method	Test CMC（%）		
		F1	Precision	Recall
**Cascade R-CNN**	——	68.0	70.1	66.0
	+ DiCoA[a]	74.2	72.8	75.8
	+ DiCoA[b]	74.1	73.3	75.0
	+ DiCoA[c]	74.3	74.3	74.4

[a] Using a receptive field size of 3 × 3 with a dilation rate of 1.
[b] Using a receptive field size of 3 × 3 with a dilation rate of 2.
[c] Using a receptive field size of 5 × 5 with a dilation rate of 1.

The DiCoA attention module can adjust its receptive field based on the size of the target, similar to the use of dilated convolution. In Table B.6, we compare the experimental results of DiCoA attention modules with different receptive field sizes. When DiCoA employs a 5 × 5 receptive field with a dilation rate of 1, compared to a 3 × 3 receptive field with a dilation rate of 1, it shows an increase of 1.5% in Precision, a decrease of 1.4% in Recall and an improvement of 0.1% in the F1. When DiCoA uses a 3 × 3 receptive field with a dilation rate of 2, compared to a 3 × 3 receptive field with a dilation rate of 1, it exhibits a 0.5% increase in Precision, with some reduction in other performance metrics. While achieving relatively good results with a 5 × 5 receptive field size using the DiCoA module, to adapt the extracted feature vectors of the network to the physical size of mitotic cells, we opted for a receptive field size of 3 × 3.

B.3. Optimization of Detection Result Thresholds

Supplementary Table B.7. Optimization of Detection Thresholds.

Detector	Method	Test CMC（%）			
		Thresh**	F1	Precision	Recall
Cascade R-CNN	——	0.89	68.0	70.1	66.0
	+ DiCoA*	0.95	72.4	73.0	72.0
	+ DiCoA	0.48	74.3	72.7	76.0

* Unupdated bounding box confidence.
** The optimal threshold.

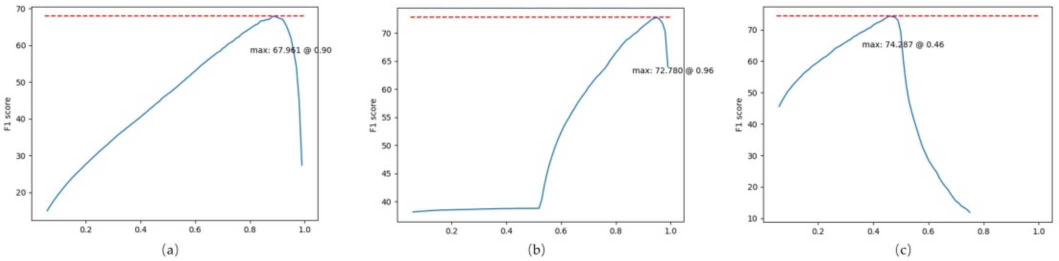

Supplementary Figure B.2. Detection result threshold optimization curves, (a) Optimization of the threshold for the Cascade R-CNN detection network. (b) Optimization of the threshold for the Cascade R-CNN with DiCoA detection network. (c) Optimization of the threshold for the Cascade R-CNN with modified DiCoA* detection network.

B.4. T-Test Results

Supplementary Table B.8. The T-test results (n=5) of our proposed DiCoA method compared to the other methods.

Our Detector	Detection algorithm	T-statistic	P-value
Cascade R-CNN + DiCoA	Faster-RCNN	59.0	4.951e-07
	Cascade R-CNN	62.0	4.042e-07
	RetinaNet	25.2	1.476e-05

We employed a one-sample t-test since we were unable to obtain the variance of the results for the methods proposed in the referenced papers.

B.5. Importance Experiment of the DiCoA Module

Supplementary Table B.9. Experimental Comparison of Different Values of α for the DiCoA Module.

Detector	Method	Test CMC（%）		
		F1	Precision	Recall
Cascade R-CNN	+ DiCoA(α = 0.1)	74.2	73.0	75.4
	+ DiCoA(α = 0.2)	74.5	74.2	74.8
	+ DiCoA(α = 0.3)	74.1	72.1	76.2
	+ DiCoA(α = 0.4)	74.7	71.8	77.9
	+ DiCoA(α = 0.5)	74.5	73.8	75.1
	+ DiCoA(α = 0.6)	73.8	71.8	75.8
	+ DiCoA(α = 0.7)	73.2	71.9	74.6
	+ DiCoA(α = 0.8)	72.5	68.2	77.5
	+ DiCoA(α = 0.9)	69.8	63.0	78.3

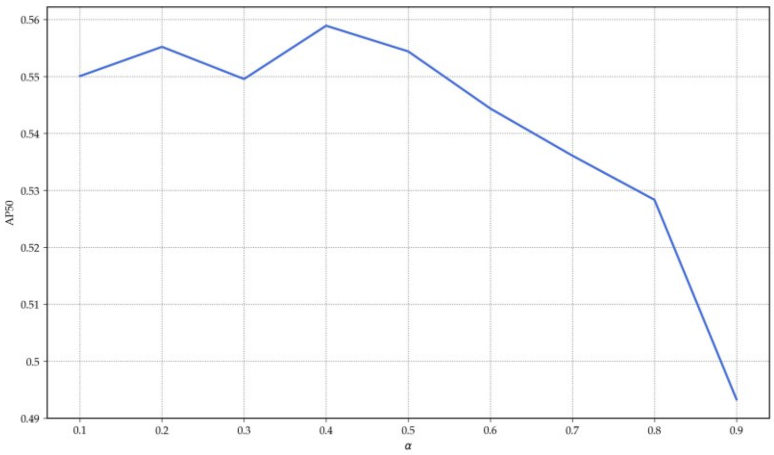

Supplementary Figure B.3 The α-AP curve illustrating the impact of the dilated contextual attention scores, P_{dicoa}, on the model.

α represents the significance of P_{dicoa}, and Figure B.3 illustrates the impact of different α values on the model through the α-AP curve. It demonstrates that the model achieves optimal performance when α is between 0.3 and 0.5. Additionally, Table B.9 explores the influence of α values on experimental results. In contrast, the model achieves a relatively better precision when α is set to 0.5 compared to 0.3 and 0.4.

B.6. Effect of *ω*

Supplementary Table B.10. The impact of ω on the performance of the method on the CMC dataset.

ω	**Test CMC（%）**		
	F1	Precision	Recall
**0**	82.4	82.5	82.4
**0.1**	82.6	82.0	83.3
**0.2**	82.7	83.8	81.7
**0.3**	82.9	83.7	82.1
**0.4**	82.9	83.3	82.5
**0.5**	82.5	83.7	81.4
**0.6**	82.2	82.5	81.8
**0.7**	81.8	82.3	81.2
**0.8**	80.8	82.3	79.3
**0.9**	79.3	81.6	77.1

We investigated the hyperparameter ω, which is used to determine the relative importance of the detection and classification stage results. We compared the effects of ω by using different configurations and measured its performance across the entire model. Table B.10~~7~~. shows that when ω is set to less than 0.5, indicating a greater emphasis on the results from the classification stage, the performance is relatively better.

B.7. Box Plot of Test Results

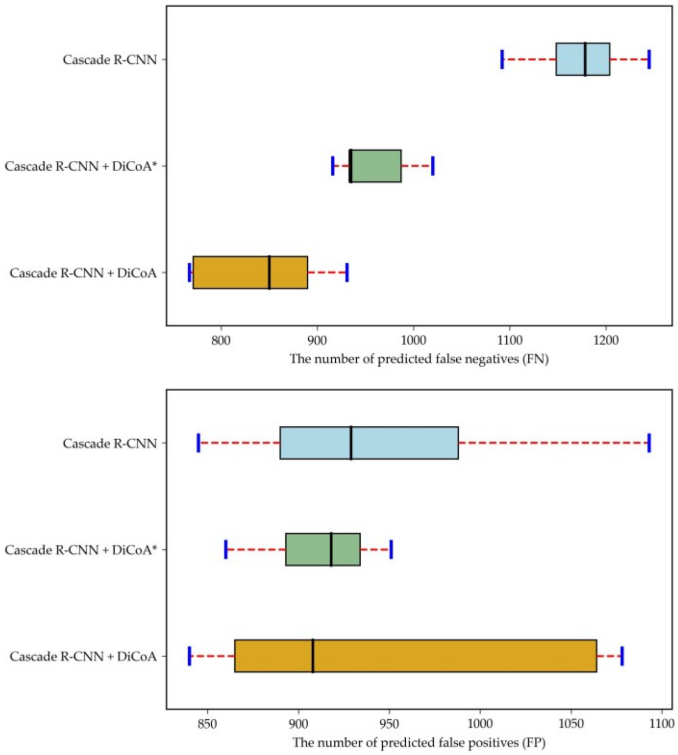

Supplementary Figure B.4. Box plots illustrating the distribution of false negative (FN) and false positive (FP) counts during testing.

B.8. Efficacy Ablation Experiments of InPreMo

Supplementary Table B.11. Single/Multi-Model Classification Experiments and Complexity Comparison.

Method	Test CMC（%）					
	F1	Precision	Recall	Params	FLOPs	MACs*
**EfficientNet-B4**	81.5	80.2	82.8	17.6M	0.9G	0.5G
**EfficientNet-B7**	82.6	83.4	81.8	63.8M	3.3G	1.6G
**Densenet121**	82.0	80.6	83.4	6.96M	1.9G	0.9G
**Densenet201**	81.9	80.5	83.5	18.1M	2.8G	1.4G
**Resnet50**	81.8	81.5	82.0	23.5M	2.5G	1.3G
**VGG16**	81.3	79.8	82.8	14.7M	10.0G	5.0G
**EfficientNet-B4 + Densenet121**	82.0	81.9	82.1	24.5M	2.8G	1.4G
**EfficientNet-B4 + Densenet201**	82.2	80.9	83.5	35.6M	3.8G	1.9G
**EfficientNet-B4 + VGG16**	82.4	83.4	81.4	32.3M	11.0G	5.5G
**EfficientNet-B7 + Densenet121**	82.1	82.5	81.8	70.7M	5.2G	2.6G
**EfficientNet-B7 + Densenet201**	81.4	82.4	80.4	81.9M	6.1G	3.1G
**EfficientNet-B7 + VGG16**	82.9	82.6	83.2	78.5M	13.3G	6.7G
**EfficientNet-B7 + Resnet50**	82.4	82.8	82.1	87.3M	5.8G	2.9G
**Densenet201 + VGG16**	80.9	80.1	81.6	32.8M	12.9G	6.4G
**Resnet50 + VGG16**	81.3	81.2	81.6	38.3M	12.6G	6.3G
**EfficientNet-B4 + Densenet121 + VGG16**	82.4	81.8	83.1	39.2M	12.8G	6.4G
**EfficientNet-B4 + Densenet201 + VGG16**	82.5	82.3	82.6	50.4M	13.8G	6.9G
**EfficientNet-B7 + Densenet121 + VGG16**	81.6	81.0	82.2	85.5M	15.2G	7.6G
**EfficientNet-B7 + Densenet201 + VGG16**	81.0	80.8	81.2	96.6M	16.1G	8.1G
**EfficientNet-B7 + Resnet50 + VGG16**	82.6	84.4	80.9	0.1G	15.8G	7.9G

* The Multiply-Accumulate Operations (MACs) quantify the computational complexity of the model.

The mitotic cells located during the detection stage were subjected to reidentification in the classification stage. Table B.8 provides comparative data on the model's classification experiments and complexity. The spatial complexity of the model is represented by the parameter count (Params), which denotes the total number of trainable parameters in the network. The temporal complexity of the model is expressed through the floating-point operation count (FLOPs) and multiply-accumulate operations (MACs), serving as metrics for the computational complexity of the model. For the integration of multiple pre-trained models in InPreMo, the time and space complexities are the sums of the respective time and space complexities of the individual pre-trained models.

In the single-model classification task, the use of the EfficientNet-B7 pre-trained model outperforms EfficientNet-B4, exhibiting a superior performance with increases of 3.2% and 1.1% in Precision and F1, respectively. On the other hand, Densenet121 performs better when compared to Densenet201 pre-trained models. However, VGG16 yields relatively poorer results compared to other pre-trained models.

When combining two pre-trained models, although the VGG16 pre-trained model performs relatively poorly in single-model experiments, the combination of EfficientNet-B7 and VGG16 pre-trained models achieves optimal results. In comparison to the combination of EfficientNet-B4 and VGG16, Precision and F1 increased by 3.2% and 0.9%, respectively, compared to EfficientNet-B4 alone, and improved by 3.6% and 1.1%, respectively, compared to VGG16. The combination of EfficientNet-B4

and Densenet201 also enhanced overall performance, while the combination of EfficientNet-B7 and Densenet201 resulted in a decrease in performance.

When combining three pre-trained models, EfficientNet-B7, Resnet50, and VGG16, we achieved relatively favorable experimental results. The most significant improvement was observed in Precision, where compared to EfficientNet-B7 alone, there was an increase of 1%, and compared to the combination of EfficientNet-B7 and VGG16, there was an increase of 1.8%. However, this performance enhancement is accompanied by a significant increase in the number of parameters.

In the classification stage, the combination of EfficientNet-B7 and Densenet121 does not outperform the performance of the individual pre-trained model EfficientNet-B7, but it surpasses the performance of the individual pre-trained model Densenet121. Simultaneously, the fusion of pre-trained models does not necessarily lead to performance improvement. The combination of EfficientNet-B7 and Densenet201 lowers the overall performance, placing it below the performance of any single pre-trained model. We observed that the combination of highly complex models often introduces adverse effects, especially for models that have exhibited overfitting. Densenet121 performs better compared to Densenet201, possibly because Densenet201 has shown signs of overfitting. The combination of highly complex models such as EfficientNet-B7 and Densenet201 results in a noticeable decrease in performance. The decline is more significant compared to the combination of EfficientNet-B7 and Densenet121, potentially due to the former utilizing the higher model complexity of EfficientNet-B7 and the overfitted Densenet201. Combining models with lower complexity can mitigate the negative impact of overfitting on model performance. For instance, the combination of EfficientNet-B4 and Densenet201, while employing the overfitted Densenet201, benefits from the relatively lower model complexity of the EfficientNet-B4 pre-trained model. Ultimately, the performance of the combination of EfficientNet-B4 and Densenet201, compared to the EfficientNet-B4 model, shows improvements of 0.7%, 0.7%, and 0.7% in Precision, Recall, and F1, respectively. Compared to the Densenet201 model, the combination exhibits improvements of 0.4% and 0.3% in Precision and F1, respectively.

B.9. Evaluation of Sensitivity and Specificity in the Classification Stage

Supplementary Table B.12. Evaluation of Sensitivity and Specificity in the Classification Stage.

**Method**	**Test CMC（%）**		
	Accuracy	Sensitivity	Specificity
**EfficientNet-B7**	90.2	91.8	85.9
**VGG16**	89.9	92.4	83.2
**EfficientNet-B7 + VGG16**	90.4	92.6	84.6
**EfficientNet-B7 + Resnet50 + VGG16**	90.2	93.0	82.7

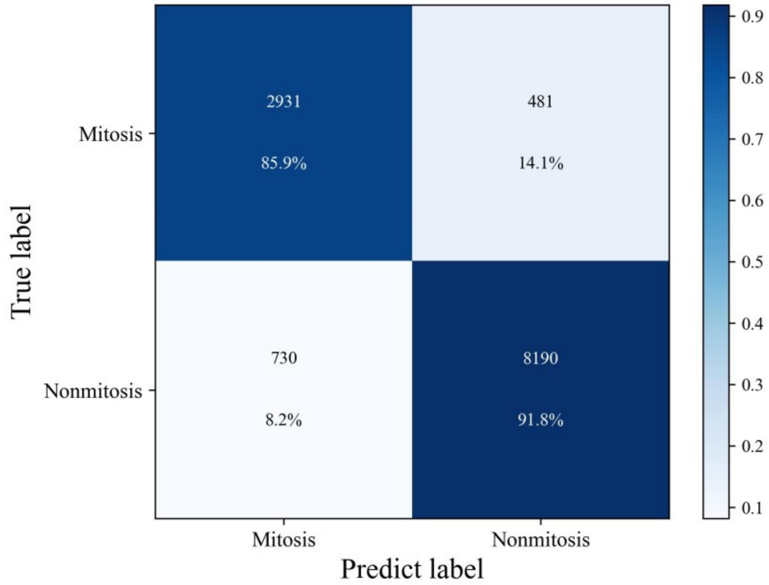

Supplementary Figure B.5. Confusion matrix for the EfficientNet-B7 model.

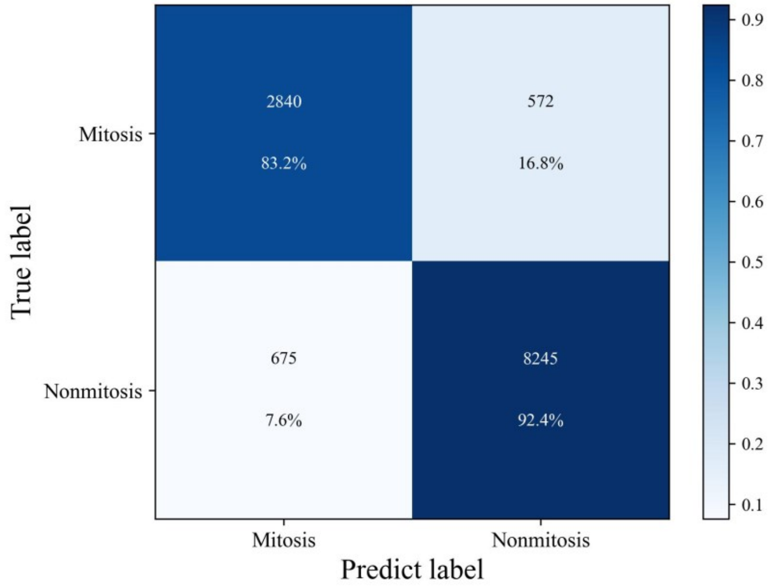

Supplementary Figure B.6. Confusion matrix for the VGG16 model.

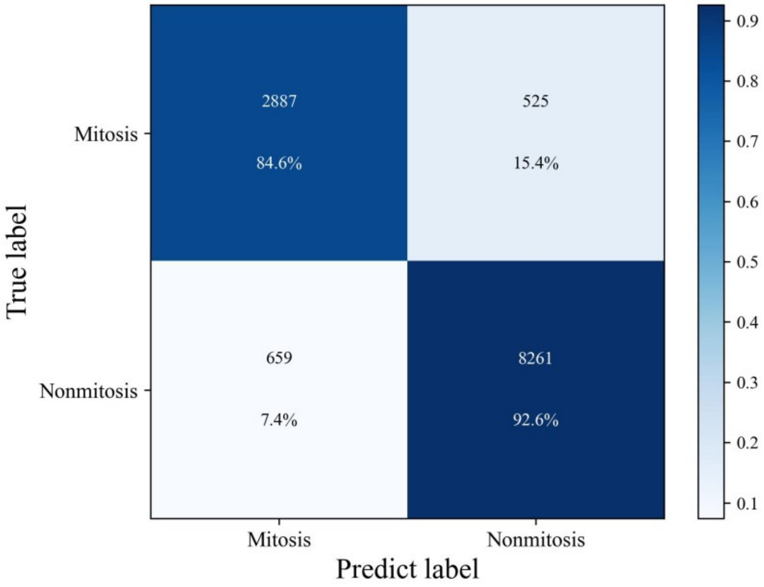

Supplementary Figure B.7. Confusion matrix for the EfficientNet-B7 and VGG16 model.

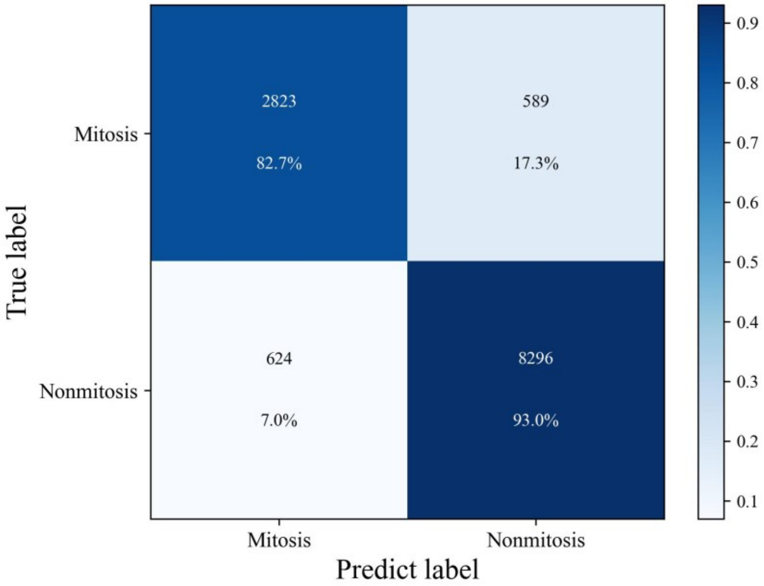

Supplementary Figure B.8. Confusion matrix for the EfficientNet-B7, Resnet50, and VsGG16 model.

B.10. The Performance Comparison of Different Networks

Supplementary Table B.13. The performance comparison of different networks on mitosis detection tasks.

Model	Task	Precision	Recall	F1 Score	Dataset	The number of mitosis	Model Information
**Our method**	Detection + Classification	82.6	83.2	82.9	CMC	13907	Cascade R-CNN combined with EfficientNet-B7 and VGG16 classification networks
		83.2	82.9	83.0	CCMCT	44880	
**D. Cireşan et al.**	Classification	88.6	70.0	78.2	ICPR 2012 MITOSIS	327	Constructing a Classifier Using Deep Neural Networks
**M. Sebai et al.**	Classification	64.4	50.7	57.5	ICPR 2014 MITOSIS	749	Constructing a Classifier Using a Semi-Supervised Deep Learning Framework
**S. Albarqouni et al.**	Classification	44.1	42.4	43.3	AMIDA13	1083	AggNet classification network incorporating training crowdsourced data
**E. Zerhouni et al.**	Classification	67.5	62.3	64.8	TUPAC16	1552	Wide Residual Network constructing a classifier
**C. Piansaddhayanaon et al.***	Detection + Classification	80.2	84.5	82.3	CMC	13907	Faster-RCNN combined with EfficientNet-B4 classification network
		83.0	83.4	83.2	CCMCT	44880	
**C. Piansaddhayanaon et al.***	Detection + Classification	82.0	81.9	81.9	CMC	13907	Cascade R-CNN combined with EfficientNet-B4 classification network
		83.2	82.6	83.0	CCMCT	44880	
**M. Aubreville et al.**	Detection + Classification	77.0	77.9	77.5	CMC	13907	RetinaNet combined with ResNet-18 classification network
		57.7	68.8	62.8	CCMCT	44880	

* The various approaches in this article.
